# Quantitative longitudinal T2* mapping for assessing placental function and association with adverse pregnancy outcomes across gestation

**DOI:** 10.1371/journal.pone.0270360

**Published:** 2022-07-19

**Authors:** Matthias C. Schabel, Victoria H. J. Roberts, Karen J. Gibbins, Monica Rincon, Jessica E. Gaffney, Aaron D. Streblow, Adam M. Wright, Jamie O. Lo, Byung Park, Christopher D. Kroenke, Kathryn Szczotka, Nathan R. Blue, Jessica M. Page, Kathy Harvey, Michael W. Varner, Robert M. Silver, Antonio E. Frias

**Affiliations:** 1 Advanced Imaging Research Center, Oregon Health and Science University (OHSU), Portland, Oregon, United States of America; 2 Division of Reproductive and Developmental Sciences, Oregon National Primate Research Center (ONPRC), OHSU, Portland, Oregon, United States of America; 3 Department of Obstetrics and Gynecology, OHSU, Portland, Oregon, United States of America; 4 Biostatistics Shared Resource, Knight Cancer Institute, OHSU, Portland, Oregon, United States of America; 5 Division of Neuroscience, ONPRC, OHSU, Portland, Oregon, United States of America; 6 Department of Obstetrics and Gynecology, University of Utah, Salt Lake City, Utah, United States of America; University of New South Wales, AUSTRALIA

## Abstract

Existing methods for evaluating *in vivo* placental function fail to reliably detect pregnancies at-risk for adverse outcomes prior to maternal and/or fetal morbidity. Here we report the results of a prospective dual-site longitudinal clinical study of quantitative placental T2* as measured by blood oxygen-level dependent magnetic resonance imaging (BOLD-MRI). The objectives of this study were: 1) to quantify placental T2* at multiple time points across gestation, and its consistency across sites, and 2) to investigate the association between placental T2* and adverse outcomes. 797 successful imaging studies, at up to three time points between 11 and 38 weeks of gestation, were completed in 316 pregnancies. Outcomes were stratified into three groups: (UN) uncomplicated/normal pregnancy, (PA) primary adverse pregnancy, which included hypertensive disorders of pregnancy, birthweight <5th percentile, and/or stillbirth or fetal death, and (SA) secondary abnormal pregnancy, which included abnormal prenatal conditions not included in the PA group such as spontaneous preterm birth or fetal anomalies. Of the 316 pregnancies, 198 (62.6%) were UN, 70 (22.2%) PA, and 48 (15.2%) SA outcomes. We found that the evolution of placental T2* across gestation was well described by a sigmoid model, with T2* decreasing continuously from a high plateau level early in gestation, through an inflection point around 30 weeks, and finally approaching a second, lower plateau in late gestation. Model regression revealed significantly lower T2* in the PA group than in UN pregnancies starting at 15 weeks and continuing through 33 weeks. T2* percentiles were computed for individual scans relative to UN group regression, and z-scores and receiver operating characteristic (ROC) curves calculated for association of T2* with pregnancy outcome. Overall, differences between UN and PA groups were statistically significant across gestation, with large effect sizes in mid- and late- pregnancy. The area under the curve (AUC) for placental T2* percentile and PA pregnancy outcome was 0.71, with the strongest predictive power (AUC of 0.76) at the mid-gestation time period (20–30 weeks). Our data demonstrate that placental T2* measurements are strongly associated with pregnancy outcomes often attributed to placental insufficiency.

**Trial registration:** ClinicalTrials.gov: NCT02749851.

## Introduction

The fundamental role played by the placenta in fetal development, pregnancy morbidity, and neonatal, pediatric, and even lifelong health is well-established [1–7]. Aberrant placental development has been linked to many adverse obstetric outcomes, including abnormalities in fetal growth, preeclampsia, preterm labor, and stillbirth [4, 8–17]. A longstanding goal of pregnancy care is to detect abnormalities in placental function prior to fetal or maternal morbidity. Obstetric imaging, predominantly with ultrasound (US), can detect fetal growth restriction (FGR) and oligohydramnios once they have occurred and is used in surveillance of pregnancies with increased risk of these conditions [18–22]. Uterine artery Doppler velocimetry has modest power to predict future severe, early onset preeclampsia and FGR prior to their occurrence [19, 21, 23], but performs poorly in predicting later onset morbidity [24]. Ultrasound observation of abnormal blood flow via umbilical artery Doppler assessment is associated with increased risk of perinatal mortality [25, 26]. However, its principal utility is in antenatal surveillance to guide hospitalization and timing of delivery after the diagnosis of FGR has already been established by ultrasound-based biometry, not in prediction of incipient FGR. There remains a need for better detection of abnormal placental function prior to clinical maternal or fetal morbidity to both optimize clinical trials and guide clinical care through appropriate treatment, and/or preparation for indicated preterm delivery.

Magnetic resonance imaging (MRI) has been used during pregnancy for decades, primarily to assess fetal abnormalities via anatomic imaging and more recently has been used to assess placental function [27]. T2* is highly sensitive to changes in the relative levels of oxyhemoglobin and deoxyhemoglobin via the blood oxygenation level dependent (BOLD) effect [28]. Early work by Sorensen and colleagues [29] observed spatial heterogeneity in T2*-weighted MRI of the placenta that was decreased by maternal hyperoxia. Our group performed the first studies that combined dynamic contrast enhanced (DCE-) MRI with quantitative T2* mapping [30, 31] in pregnant nonhuman primates (NHPs), demonstrating that the heterogeneity in placental T2* arises from spatial gradients in intervillous maternal placental blood (MPB) oxygen saturation within functional lobules. Since this early animal work, placental T2* has been measured in several small pilot studies in both uncomplicated human pregnancies and human pregnancies with adverse outcomes [32–39], verifying the decreasing trend with gestational age observed in NHP studies. These human studies also found reduced placental T2* and reduced placental perfusion fraction in pregnancies complicated by small for gestational age [40–42] in preterm preeclampsia. Recent NHP studies from our group have further shown that anomalous baseline T2* values are correlated with placental dysfunction in cases of FGR [43] and are also noted with prenatal alcohol exposure [44] or maternal Zika virus infection [45].

The primary objectives of this work were (a) to establish a reference data set for the longitudinal evolution of placental T2* across gestation in uncomplicated pregnancies, (b) to analyze a large enough group of pregnancies to determine predictive power of T2* prior to onset of clinical morbidity, and (c) to compare findings between sites in order to assess generalizability across different institutions using compatible scanner hardware and MRI protocols and analyzed by uniform postprocessing. A secondary objective was to assess the impact of various complications of pregnancy on placental T2* values.

## Materials and methods

### Study design

This is a longitudinal prospective study of 316 pregnant women at two sites, both academic tertiary care centers with Level IV neonatal intensive care units. (ClinicalTrials.gov: NCT02749851). Participants were recruited from the Oregon Health & Science University (OHSU) and University of Utah Health Sciences Center (UU) clinics with IRB approval at both sites. The two study sites have similar patient demographics but notable difference in altitude (OHSU is located at 450 feet above sea level, the University of Utah at 4,840 feet above sea level), an environmental exposure that can alter hemoglobin and SpO_2_ levels and, potentially, placental T2*. With our goal of evaluating generalizability of this metric, we sought to test it under different environmental conditions.

### Inclusion criteria

We recruited both “low-risk” and “high-risk” individuals with the goal of ensuring sufficient adverse outcome numbers in the study by intentional enrichment with “high-risk” pregnancies. Inclusion criteria for both groups were: pregnancy (defined by positive pregnancy test and certain menstrual history, or early ultrasound) identified prior to 16 weeks gestation, maternal age over 18 years of age, and ability to give informed consent. Inclusion criteria for the low-risk group included: 1) no history of second or third trimester pregnancy loss, 2) no history of FGR or small for gestational age (SGA), and 3) nonsmoker. Inclusion criteria for the high-risk group were one or more of the following: 1) history of previous singleton pregnancy complicated by preeclampsia with severe features requiring preterm delivery, or preterm delivery due to placental insufficiency (FGR, oligohydramnios, abnormal umbilical artery Doppler), or SGA with neonatal weight < 10th percentile delivered at term, or stillbirth attributed to placental cause, regardless of gestational age, 2) pregnancy at risk for placental insufficiency due to clinical comorbidities (e.g. chronic hypertension, pre-gestational diabetes, systemic lupus erythematosus, renal disease), or 3) history of spontaneous preterm birth < 34 weeks. Although spontaneous preterm birth is not classically considered a presentation of placental insufficiency, we included them in our high-risk group because histopathology in placentas from spontaneous preterm births demonstrate maternal vascular malperfusion lesions at similar frequency as indicated preterm births [46]. All pregnancy management was at the discretion of the participant’s provider, including whether or not to recommend treatment with low dose aspirin. It is the practice of both participating sites to recommend low dose aspirin for individuals at increased risk of preeclampsia, according to the American College of Obstetricians and Gynecologists (ACOG) [47].

### Exclusion criteria

Exclusion criteria were maternal intellectual disability or incarceration, pregnancy with major fetal anomalies known to be associated with abnormal fetal growth, active alcohol use during pregnancy, medical conditions requiring ongoing treatment during pregnancy including cancer, acute liver disease, chronic pulmonary disease requiring regular use of medication, history of claustrophobia, metal implants, or other contraindication for MRI, and increased risk of aneuploidy based on ultrasound findings and/or genetic testing.

### Participant enrollment

Informed consent was obtained from all participants. The original study protocol planned enrollment of 300 participants to undergo three sequential MRI studies in the following gestational windows: 12–16 weeks, 26–28 weeks, and 32–34 weeks. The rationale for this study design was to facilitate characterization of T2* longitudinally during pregnancy and to minimize sensitivity to population variability in T2* values as a function of gestational age. A planned interim analysis in year 3 demonstrated strong correlation of T2* across study sites and within gestational age timepoints. As a result, the decision was made to expand the gestational time windows for recruitment to cover an overlapping range from 10 to 40 weeks, allowing us to characterize evolution of T2* as a continuous variable. Histograms of number of MRI studies performed by gestational week are plotted in S1a Fig (stratified by category) and S1b Fig (stratified by site).

### Pregnancy outcome designation

Pregnancy outcomes were categorized as follows: uncomplicated/normal (UN), primary adverse outcome (PA), and secondary abnormal outcome (SA). UN pregnancies were defined as those with term delivery (37 weeks or beyond) and birthweight between the 5th and 95th percentile, without gestational hypertensive disease, and not meeting any other criteria for the primary adverse or secondary abnormal outcomes. The primary adverse outcome group (PA) was defined as a composite including hypertensive disorders of pregnancy, small for gestational age defined as birthweight below the 5th percentile per Oken birthweight tables [48], and/or stillbirth or fetal death. Hypertensive disorders of pregnancy included gestational hypertension, preeclampsia (with or without severe features), HELLP (hemolysis, elevated liver enzymes, and low platelet count) syndrome, or eclampsia, as defined by ACOG criteria [47].

The secondary abnormal outcome group (SA) included pregnancies that did not meet criteria for PA but were still complicated. This group included maternal chronic hypertension without superimposed preeclampsia, fetal genetic and/or anatomic anomalies; spontaneous preterm birth due to preterm labor, cervical insufficiency, and/or preterm premature rupture of the membranes (PPROM); placental abruption, chorioamnionitis (as diagnosed by delivering provider), and/or birthweight greater than the 95th percentile by Oken [48]. Adjudication of the PA outcomes and SA outcomes was performed independently by two board-certified Maternal-Fetal Medicine physicians from each site (OHSU: KJG, AEF; UU: JMP, NRB). Any discordance between assessment of outcomes was then discussed and reconciled prior to final determination. The authors determining the outcomes were blinded to the MRI data and analysis prior to adjudication of clinical outcome group. Histograms of total enrollment by site and group are shown in S1a and S1b Fig.

### Magnetic resonance imaging

MRI in pregnant participants was performed at both sites using identical imaging protocols on 3T Siemens Prisma scanner hardware using vendor spine and body array coils. Following localization of the uterus and placenta and acquisition of T2-HASTE anatomic imaging in three planes (axial, coronal, and sagittal), breath-hold multi-slice multi-echo gradient echo (MEGE) images were acquired in an axial orientation for T2* mapping, spanning the entire uterus with a spatial resolution of 1.75x1.75x3.5 mm, at six in-phase echo times (TE): 4.92 ms, 9.84 ms, 19.68 ms, 29.52 ms, 36.90 ms, and 49.20 ms with a repetition time (TR) of 116 ms. Number of slices and in-plane field-of-view were adjusted as necessary to achieve complete coverage of the uterus and avoid image wrap. Breath-hold duration was maintained below 10 seconds per acquisition to minimize patient discomfort. In the OHSU studies only, 3D variable flip angle T1 mapping was also performed with full placental coverage, using the Siemens MapIt protocol with flip angles of 3 and 15 degrees, including B1 correction (resolution 0.9x0.9x4 mm, TR = 5.01 ms, TE = 2.23 ms). Maternal blood draws were performed prior to each scan and hemoglobin level measured using iStat (Abbott, Princeton, NJ) and/or fingerstick. Pulse oximetry (Zacurate 500BL) was used to determine maternal blood oxygen saturation level before each MRI study. MRI data acquisition, post-processing, region of interest (ROI) designation, and quality control were blinded to pregnancy outcome group. Placental ROIs were drawn by research assistants in our laboratory and individually reviewed by an MRI physicist with eight years of experience in placental MRI (MCS). Binary masks were derived from the placental ROIs, with T2* values of 250 ms or more being excluded from further analysis as these large values are associated with signal contamination by amniotic fluid. Placental volume was computed by summing the number of voxels in each slice of this binary mask multiplied by the per-voxel volume. Where slices were missing due to motion, volumes were estimated from adjacent slices using linear interpolation. In the OHSU cohort, median placental T1 was determined by spatially resampling measured T1 maps onto the T2* image volumes and applying placental ROIs.

### Data analysis and statistics

Specific power calculations were not made due to the exploratory nature of our investigation. All p-values for continuous variables presented in this manuscript were computed using a two-tailed Kolmogorov-Smirnov test, and effect sizes were computed using Cohen’s d. The chi-square proportion test was used to compare frequencies of binary outcomes. Distributions of T2* within the placenta are notably non-Gaussian, so placental average values of T2* were computed using the median. Continuous data modeling of measured variables vs. gestational age was performed using nonlinear least squares regression, with the Bayes Information Criterion used to guide model selection among a group of plausible candidate models comprised of (1) constant: *y*(*t*) = *p*_1_, (2) linear: *y*(*t*) = *p*_1_ + *p*_2_^*t*^, (3) quadratic: *y*(*t*) = *p*_1_ + *p*_2_*t* + *p*_3_*t*^2^, (4) cubic: *y*(*t*) = *p*_1_ + *p*_2_*t* +*p*_3_*t*^2^ + *p*_4_*t*^3^, and (5) sigmoid: *y*(*t*) = *p*_1_/(1 + *exp* (*p*_2_ (*t* − *p*_3_))) + *p*_4_. We chose a logistic sigmoid function as it is commonly used for this type of modeling. Modeling of the temporal rates of change within individual pregnancies was performed using the analytical time derivative of the corresponding continuous model. The significance of differences in regressions was assessed using the presence or absence of overlapping 95% confidence intervals from modeled covariance. Z-scores were computed using the regression model for UN pregnancies, along with the modeled confidence intervals. Receiver operating characteristic (ROC) curves were developed using these z-scores to assess the test characteristics of placental T2* for PA or SA outcomes, and c-statistics were reported. These were created for both the entire population, and stratified by time window in gestation, and by site. Gestational time windows were 10–20 weeks, 20–30 weeks, and 30+ weeks. Due to relatively low numbers enrolled within the high-risk group, the data and analysis does not attempt to stratify on low-risk vs. high-risk enrollment. All data and software needed to reproduce the results presented in this manuscript are available in open source form (https://github.com/matthiasschabel/OHSU-U01-HPP-2021).

### Study approval

All protocols described in the following were approved by the Institutional Review Boards (IRB) at OHSU and UU, and study oversight was provided by an independent data and safety monitoring board. Participants were fully informed of study procedures and written consent was obtained at the time of enrollment.

## Results

### Participant and study demographics

**Fig 1:**
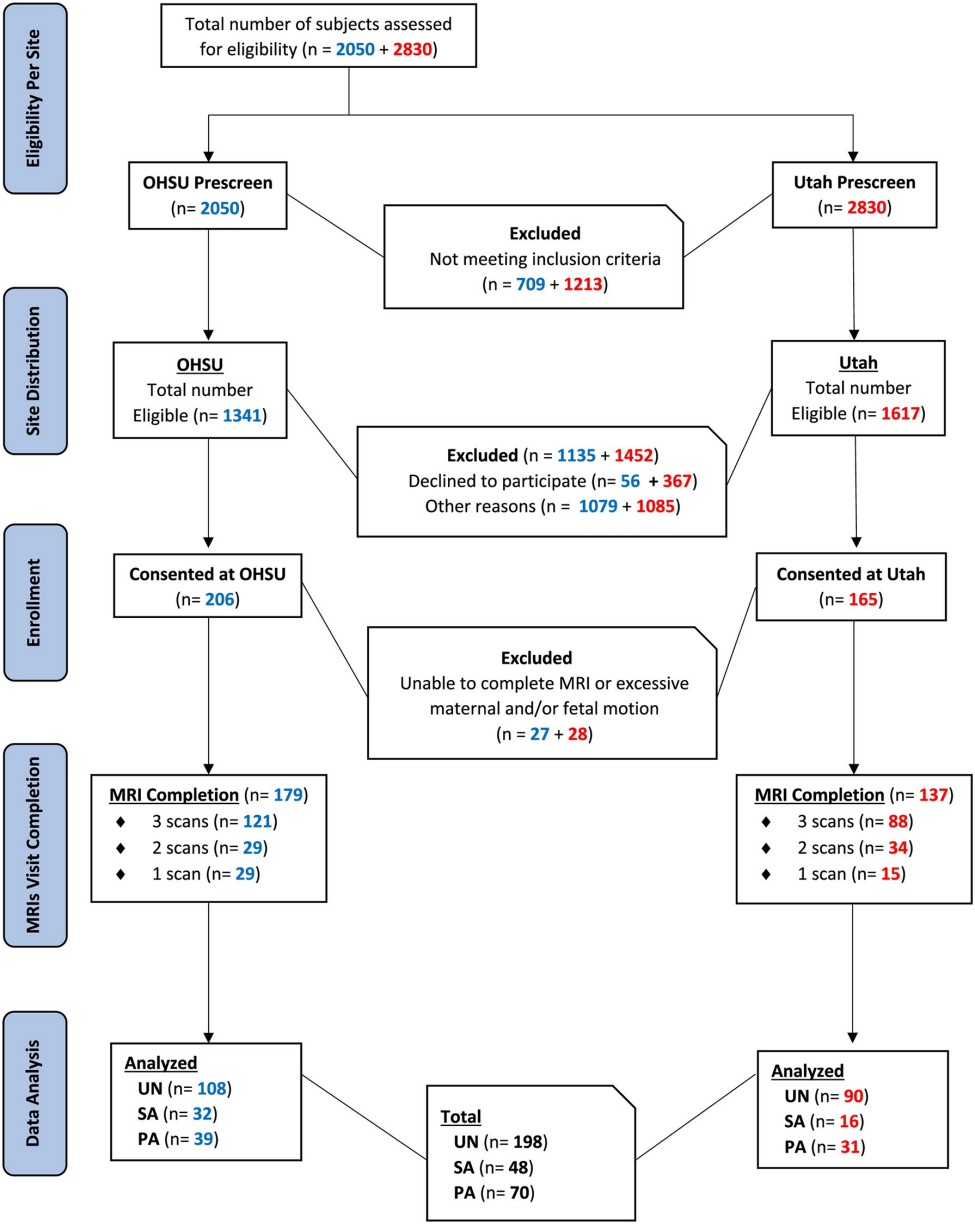
Enrollment flow chart. Numbers detail prospective patients screened, consented, and enrolled at both study sites, along with completed MRI studies meeting quality criteria for inclusion in data analysis presented here. The majority of exclusions were due to lack of child care required to attend study visits, marijuana use and/or concomitant medical conditions.

Details of participant enrollment and study completion at the two study sites are presented in the flow chart in Fig 1. Demographics and maternal characteristics are detailed in Table 1. Tobacco use was more common in participants from the OHSU site than from the Utah site (14.0% vs 6.6%, RR 2.11 [95% CI 1.02–4.37]). MRI data of adequate quality to perform T2* analysis were acquired in 797 imaging studies from 316 individual study participants (450 scans from 179 participants at OHSU, 347 scans from 137 participants at Utah). At least one complete MRI scan of sufficient quality for T2* analysis (e.g., no motion artifact) was obtained from 86% of participants who were consented (88% at OHSU, 83% at Utah), with all three scans completed in 66% of these patients, two scans in 20%, and a single scan in 14%. Of these studies, 700 had usable hemoglobin data (426 at OHSU, 274 at Utah) and 432 had usable SpO_2_ measurements (252 at OHSU, 180 at Utah).

**Table 1:** Demographic data from the study populations at OHSU and UU.

	UN			PA			SA		
	All	OHSU	Utah	All	OHSU	Utah	All	OHSU	Utah
**Maternal age**	31.2 (4.6)	31.8 (5.1)	30.4 (4.0)	31.7 (5.1)	32.2 (5.3)	31.0 (4.9)	31.0 (5.4)	30.9 (5.7)	31.4 (4.7)
**Race**									
White	175 (81.4%)	97 (79.5%)	78 (83.9%)	55 (75.3%)	29 (69.0%)	26 (83.9%)	42 (73.7%)	27 (69.2%)	15 (83.3%)
African Descent	9 (4.2%)	9 (7.4%)	0 (0.0%)	1 (1.4%)	1 (2.4%)	0 (0.0%)	2 (3.5%)	1 (2.6%)	1 (5.6%)
Native American	5 (2.3%)	3 (2.5%)	2 (2.2%)	0 (0.0%)	0 (0.0%)	0 (0.0%)	3 (5.3%)	2 (5.1%)	1 (5.6%)
Asian Indian	4 (1.9%)	3 (2.5%)	1 (1.1%)	1 (1.4%)	0 (0.0%)	1 (3.2%)	0 (0.0%)	0 (0.0%)	0 (0.0%)
Other Asian	10 (4.7%)	6 (4.9%)	4 (4.3%)	6 (8.2%)	4 (9.5%)	2 (6.5%)	2 (3.5%)	2 (5.1%)	0 (0.0%)
Native Hawaiian	0 (0.0%)	0 (0.0%)	0 (0.0%)	0 (0.0%)	0 (0.0%)	0 (0.0%)	1 (1.8%)	1 (2.6%)	0 (0.0%)
Pacific Islander	0 (0.0%)	0 (0.0%)	0 (0.0%)	1 (1.4%)	0 (0.0%)	1 (3.2%)	1 (1.8%)	1 (2.6%)	0 (0.0%)
Other	1 (0.5%)	1 (0.8%)	0 (0.0%)	0 (0.0%)	0 (0.0%)	0 (0.0%)	0 (0.0%)	0 (0.0%)	0 (0.0%)
Unknown	1 (0.5%)	1 (0.8%)	0 (0.0%)	0 (0.0%)	0 (0.0%)	0 (0.0%)	0 (0.0%)	0 (0.0%)	0 (0.0%)
Hispanic	10 (4.7%)	2 (1.6%)	8 (8.6%)	9 (12.3%)	8 (19.0%)	1 (3.2%)	6 (10.5%)	5 (12.8%)	1 (5.6%)
**Pre-pregnancy BMI**	24.4 (4.4)	24.7 (4.8)	24.0 (3.9)	26.3 (5.9)	27.4 (6.5)	24.8 (4.8)	26.7 (5.8)	27.2 (6.2)	25.4 (4.6)
**Tobacco use**	13 (6.6%)	9 (8.3%)	4 (4.5%)	13 (18.6%)	10 (25.6%)	3 (9.7%)	8 (16.7%)	6 (18.8%)	2 (12.5%)

### Pregnancy outcomes

Out of 316 participants, 198 (62.6%) were uncomplicated pregnancies (UN), 70 (21.8%) resulted in primary adverse (PA) outcomes, and 48 (15.2%) resulted in secondary abnormal (SA) outcomes, as defined in the Methods. Of those enrolled in the low-risk group, 12.5% resulted in PA pregnancies, 15.8% were SA, and 71.7% were UN. Of those enrolled in the high-risk group, 46.0% resulted in PA pregnancies, 27% were SA, and 27.0% were UN. Within the PA outcome group, the most commonly observed component was preeclampsia with severe features (40.0%) followed by gestational hypertension (31.4%), and SGA (20.0%) (Table 2). Severe preeclampsia was more prevalent in the OHSU cohort than the UU cohort, but the observed differences were not statistically significant. 35.7% of those with the PA outcome delivered prior to 37 weeks. The SA group included 35.4% with spontaneous preterm birth, 14.6% with chronic HTN, 25% with fetal genetic or anatomic anomalies, 4.2% with placental abruption, 12.5% with chorioamnionitis, and 12.5% with birthweight >95th percentile.

**Table 2:** Breakdown of prenatal conditions in the primary adverse outcome group for entire PA population and by site.

Outcome	Total	OHSU	Utah	p-value
**Total PA**	70	39	31	
**PIH**	59 (84.3%)	34 (87.2%)	25 (80.6%)	0.68
**Gestational HTN**	22 (31.4%)	13 (33.3%)	9 (29.0%)	0.90
**Pre-eclampsia w/o severe features**	9 (12.8%)	3 (7.7%)	6 (19.4%)	0.28
**Pre-eclampsia with severe features**	28 (40.0%)	18 (46.2%)	10 (32.2%)	0.35
**SGA <5**^**th**^ **percentile**	11 (15.7%)	6 (15.4)	5 (16.1)	0.74
**Stillbirth or fetal loss**	4 (5.7%)	1 (2.6%)	3 (9.7%)	0.45
**Placental abruption**	2 (2.8%)	0 (0.0%)	2 (6.4%)	0.38
**Both PIH + SGA**	5 (7.1%)	3 (7.7%)	2 (6.4%)	0.79
**Preterm birth (<37w)**	31 (35.7%)	13 (33.3%)	12 (38.7%)	0.83

### Birthweight percentile

Table 3 presents statistics on birthweight percentile, along with several other physiological variables (pre-pregnancy BMI, BMI at delivery, and maternal age), stratified by site and pregnancy category. Median birthweight percentile was 48.2 in UN pregnancies, 32.7 in PA pregnancies, and 48.8 in the SA pregnancies overall. There was a significant difference, with medium effect size, in birthweight percentile between UN and PA outcome groups (p = 0.017, d = 0.43) but not between UN and SA (p = 0.28). When stratified by study site, median birthweight percentiles for UN, PA, and SA were 50.5, 30.3, and 49.3 at OHSU (with a significant difference between UN and PA, p = 0.023, d = 0.58), and 42.0, 36.1, and 46.5 at UU (non-significant).

**Table 3:** Physiological variables, with p-values and effect sizes (Cohen’s d), for study subjects stratified by category and site. Effect size is considered small (green cells) when p< = 0.05 and 0.2< = d<0.5, and medium (orange cells) when p< = 0.05 and 0.5< = d<0.8.

	median		p-value	Cohen d	median		p-value	Cohen d	median		p-value	Cohen d
	Both sites											
	UN vs. PA				UN vs. SA				PA vs. SA			
Birthweight percentile	48.19	32.70	0.017	0.43	48.19	48.84	0.276	0.02	32.70	48.84	0.089	0.41
Pre-pregnancy BMI	23.62	24.81	0.049	0.25	23.62	25.83	0.080	0.50	24.81	25.83	0.913	0.17
Delivery BMI	29.01	30.54	0.123	0.35	29.01	30.85	0.256	0.40	30.54	30.85	0.878	0.06
Maternal age	31.00	32.00	0.872	0.22	31.00	31.50	1.000	0.11	32.00	31.50	0.990	0.13
	OHSU only											
	UN vs. PA				UN vs. SA				PA vs. SA			
Birthweight percentile	50.48	30.26	0.023	0.58	50.48	49.29	0.742	0.04	30.26	49.29	0.064	0.53
Pre-pregnancy BMI	24.07	25.03	0.120	0.19	24.07	26.06	0.247	0.43	25.03	26.06	0.976	0.15
Delivery BMI	28.97	30.57	0.094	0.36	28.97	31.81	0.046	0.64	30.57	31.81	0.775	0.23
Maternal age	32.50	33.00	0.787	0.11	32.50	32.00	0.974	0.11	33.00	32.00	0.872	0.27
	Utah only											
	UN vs. PA				UN vs. SA				PA vs. SA			
Birthweight percentile	41.99	36.09	0.423	0.16	41.99	46.46	0.509	0.12	36.09	46.46	0.827	0.23
Maternal SpO_2_	97.00	96.00	0.619	0.45	97.00	96.50	0.989	0.34	96.00	96.50	0.317	0.22
Delivery BMI	29.10	29.75	0.973	0.14	29.10	28.66	0.794	0.09	29.75	28.66	0.589	0.23
Maternal age	30.00	31.50	0.610	0.37	30.00	31.00	0.956	0.22	31.50	31.00	1.000	0.10
	OHSU vs. Utah											
	UN				PA				SA			
Birthweight percentile	50.48	41.99	0.626	0.24	30.26	36.09	0.743	0.15	49.29	46.46	0.780	0.07
Pre-pregnancy BMI	24.07	23.33	0.710	0.17	25.03	23.95	0.413	0.17	26.06	25.19	0.781	0.15
Delivery BMI	28.97	29.10	0.830	0.03	30.57	29.75	0.158	0.15	31.81	28.66	0.252	0.50
Maternal age	32.50	30.00	0.007	0.56	33.00	31.50	0.657	0.40	32.00	31.00	0.958	0.18

### Placental T2*

**Fig 2:**
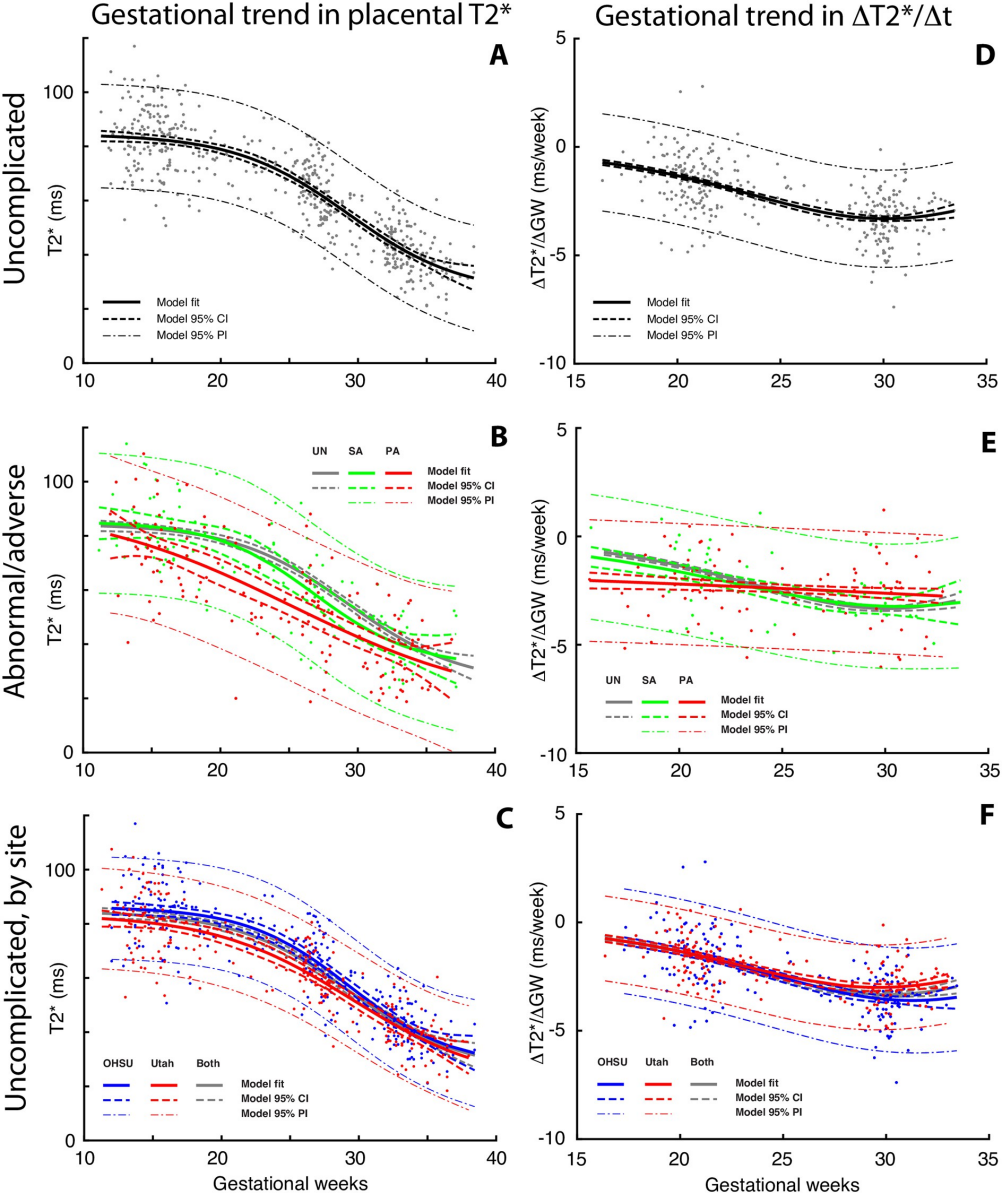
Gestational dependence of placental T2* values and rates of change. Median T2* values for each completed study, computed over the entire placenta, are plotted as a function of gestational age at time of imaging in the three panels in the left column (panels A, B, C). Rates of change in placental T2* between repeated imaging time points within the same individual, where the x value is the mean gestational age of two sequential scans and the y value is the change in T2* between the same two sequential scans, are plotted as a function of mean gestational age in the right column (panels D, E, F). The upper row plots these quantities for normal pregnancies, the middle row for abnormal (green) and adverse (red) pregnancies, and the bottom row for normal pregnancies stratified by site (OHSU in blue, Utah in red). In all graphs, model regression curves (using the functions and parameters given in Table 4) are indicated by the thick solid lines, the 95% confidence intervals by the dashed lines, and the 95% prediction intervals by the dot-dashed lines. The best fit and 95% CI curves from the UN population are superimposed in gray on the PA/SA and site-specific plots in the second and third rows for reference.

Fig 2A shows the measured dependence of placental T2* across gestation in UN pregnancies. This quantity decreases continuously throughout pregnancy, beginning at a relatively high plateau level early in gestation, then dropping increasingly rapidly to an inflection point around 30 weeks before approaching a second, lower plateau in late gestation. Sigmoid model regression was statistically-preferred relative to the other polynomial candidate models based on the Bayes Information Criterion. Table 4 provides regression model definitions and model parameters. For the PA outcome group the regression model found significantly lower predicted T2* than UN pregnancies starting at 15 weeks and continuing through 33 weeks gestation. The model fit for the SA outcome group was not significantly different from that for UN pregnancies at any point in gestation, where significance of differences in model regressions was determined based on non-overlapping 95% confidence intervals.

**Table 4:** Regression models, best fit parameter values and estimated parameter uncertainties, and root-mean-square (RMS) fit residual values for model fits of gestational trends in T2*, ΔT2*/ΔGW, maternal hemoglobin, maternal blood oxygen saturation, in vivo placental volume, and T1. Fits are presented for the aggregate UN data set along with separate regressions to the OHSU and UU UN subpopulations for normal pregnancies, and for PA and SA subgroups.

	p_1_	*p*_2_	*p*_3_	*p*_4_	*RMS*_*fit*_
Median T2* (ms)	$T_{2}^{*}\left(t\right)=p_{1}/\left(1+exp\left(p_{2}\left(t-p_{3}\right)\right)\right)+p_{4}$				
UN pregnancies	-59.2 (+/-6.5)	-0.24 (+/-0.04)	29.3 (+/-0.8)	84.6 (+/-1.3)	+/-9.6 ms
OHSU only	-59.3 (+/-7.6)	-0.26 (+/-0.05)	29.4 (+/-0.9)	86.3 (+/-1.5)	+/-9.5 ms
Utah only	-62.8 (+/-13.9)	-0.20 (+/-0.06)	29.5 (+/-1.8)	83.6 (+/-2.8)	+/-9.2 ms
SA pregnancies	-53.7 (+/-11.3)	-0.28 (+/-0.11)	26.8 (+/-1.3)	85.4 (+/-4.0)	+/-12.5 ms
PA pregnancies	-57.6 (+/-22.9)	-0.19 (+/-0.11)	25.2 (+/-2.0)	82.9 (+/-10.7)	+/-13.8 ms
ΔT2*/Δ**GW (ms/wk)**	$\Delta T_{2}^{*}/\Delta t\left(t\right)=-p_{1}p_{2}exp\left(p_{2}\left(t-p_{3}\right)\right)/{\left(1+\text{exp}\left(p_{2}\left(t-p_{3}\right)\right)\right)}^{2}$				
UN pregnancies	-64.9 (+/-4.2)	-0.20 (+/-0.01)	30.1 (+/-0.6)		+/-1.2 ms/wk
OHSU only	-75.5 (+/-9.5)	-0.19 (+/-0.02)	31.3 (+/-1.1)		+/-1.0 ms/wk
Utah only	-60.2 (+/-5.0)	-0.20 (+/-0.02)	29.6 (+/-0.7)		+/-1.1 ms/wk
SA pregnancies	-79.0 (+/-22.0)	-0.16 (+/-0.04)	30.7 (+/-2.6)		+/-1.4 ms/wk
PA pregnancies	-5574.6 (+/-1e6)	-0.02 (+/-0.24)	225.9 (+/-2e5)		+/-1.4 ms/wk
**[Hb] (mg/dl)**	[*Hb*](*t*) = *p*_1_ + *p*_2_^*t*^				
UN pregnancies	13.27 (+/-0.15)	-0.046 (+/-0.006)			+/-1.02 mg/dl
OHSU only	12.86 (+/-0.17)	-0.047 (+/-0.007)			+/-0.86 mg/dl
Utah only	13.78 (+/-0.21)	-0.042 (+/-0.008)			+/-0.90 mg/dl
SA pregnancies	12.65 (+/-0.32)	-0.018 (+/-0.013)			+/-0.92 mg/dl
PA pregnancies	13.46 (+/-0.31)	-0.039 (+/-0.012)			+/-1.04 mg/dl
**SpO**_**2**_ **(%)**	*SpO*_2_(*t*) = *p*_1_ + *p*_2_*t*				
UN pregnancies	97.05 (+/-0.57)	0.003 (+/-0.021)			+/-2.4%
OHSU only	98.37 (+/-0.38)	-0.010 (+/-0.014)			+/-1.2%
Utah only	95.24 (+/-1.03)	0.031 (+/-0.037)			+/-3.0%
SA pregnancies	98.54 (+/-0.65)	-0.043 (+/-0.025)			+/-1.4%
PA pregnancies	95.99 (+/-1.16)	0.052 (+/-0.044)			+/-3.1%
**Placental vol. (cm^3)**	*V*(*t*) = *p*_1_ + *p*_2_*t*				
UN pregnancies	-372.4 (+/-17.6)	32.2 (+/-0.68)			+/-122 cm^3
OHSU only	-377.0 (+/-25.9)	32.4 (+/-0.99)			+/-130 cm^3
Utah only	-367.7 (+/-23.6)	32.1 (+/-0.91)			+/-113 cm^3
SA pregnancies	-383.0 (+/-53.5)	33.1 (+/-2.18)			+/-158 cm^3
PA pregnancies	-360.5 (+/-35.7)	30.0 (+/-1.40)			+/-132 cm^3
**Median T1 (ms)**	*T*_1_(*t*) = *p*_1_ + *p*_2_*t*				
UN pregnancies	---	---			
OHSU only	2513 (+/-49.3)	-26.9 (+/-1.88)			+/-208 ms
Utah only	---	---			
SA pregnancies	2556 (+/-83.0)	-26.5 (+/-3.37)			+/-158 ms
PA pregnancies	2591 (+/-102.0)	-31.2 (+/-3.87)			+/-246 ms

Site-dependent data and regressions for UN pregnancies are shown in Fig 2C for OHSU (blue) and UU (red), with fit and 95% CI for all UN again plotted in gray. While the resulting curves are quite similar in shape, the Utah T2* data for UN pregnancies are consistently lower than the corresponding OHSU data, and the difference between the two is statistically significant between 15 and 29 weeks of gestation. The observed site-specific differences between T2* in UN placentas can be understood based on corresponding site differences in maternal hemoglobin and SpO_2_ levels arising from the difference in altitude between our sites (OHSU at 450 feet above sea level, and the University of Utah at 4840 feet above sea level), discussed below, and the known dependence of MRI signal on deoxyhemoglobin concentration. Hemoglobin levels are physiologically increased in those chronically at elevated altitude as an adaptation to low partial pressure of oxygen [49].

Median voxel-level relative measurement uncertainty in placental T2* data for UN pregnancies was ±7.0%, was comparable in both SA (±6.1%) and PA (±6.2%) pregnancies, and was significantly higher in the Utah studies than at OHSU (±5.8% for OHSU, 10.3% for Utah, p<0.001). In addition to stratifying based on pregnancy outcome and study site, the dependence of gestational T2* measurements in UN pregnancies on fetal sex (S2a Fig), maternal age (S2b Fig), and maternal body mass index (BMI, S2c Fig) was evaluated, with no significant differences among any of these. S2d Fig plots T2* for PA pregnancies at OHSU vs. Utah, S2e Fig plots T2* based on severity of PA features, and S2f Fig plots T2* for normal vs. low birthweight pregnancies. Exclusion of measurements not meeting heuristic data quality criteria did not significantly alter any reported results.

The average rate of change in placental T2* with gestation, computed from the centered finite difference of paired measurements in each individual pregnancy at successive time points, is plotted for UN pregnancies in Fig 2D, for PA (red), and SA (green) pregnancies in Fig 2E, and for OHSU (blue) vs. Utah (red) UN in Fig 2F. Model regressions to these data using the time derivative of the logistic function are displayed as in Fig 2A–2C. As with the T2* data themselves, the rate of change data for UN and SA pregnancies are not significantly different at any point during gestation. In contrast, the rate of change in PA pregnancies is nearly constant and shows a significantly larger rate of decrease in early and mid-gestation (up to 24 weeks) relative to UN. The rate of T2* decrease with gestation was found to be slightly, but significantly, larger in OHSU versus Utah UN pregnancies from 28 weeks gestation onward.

**Fig 3:**
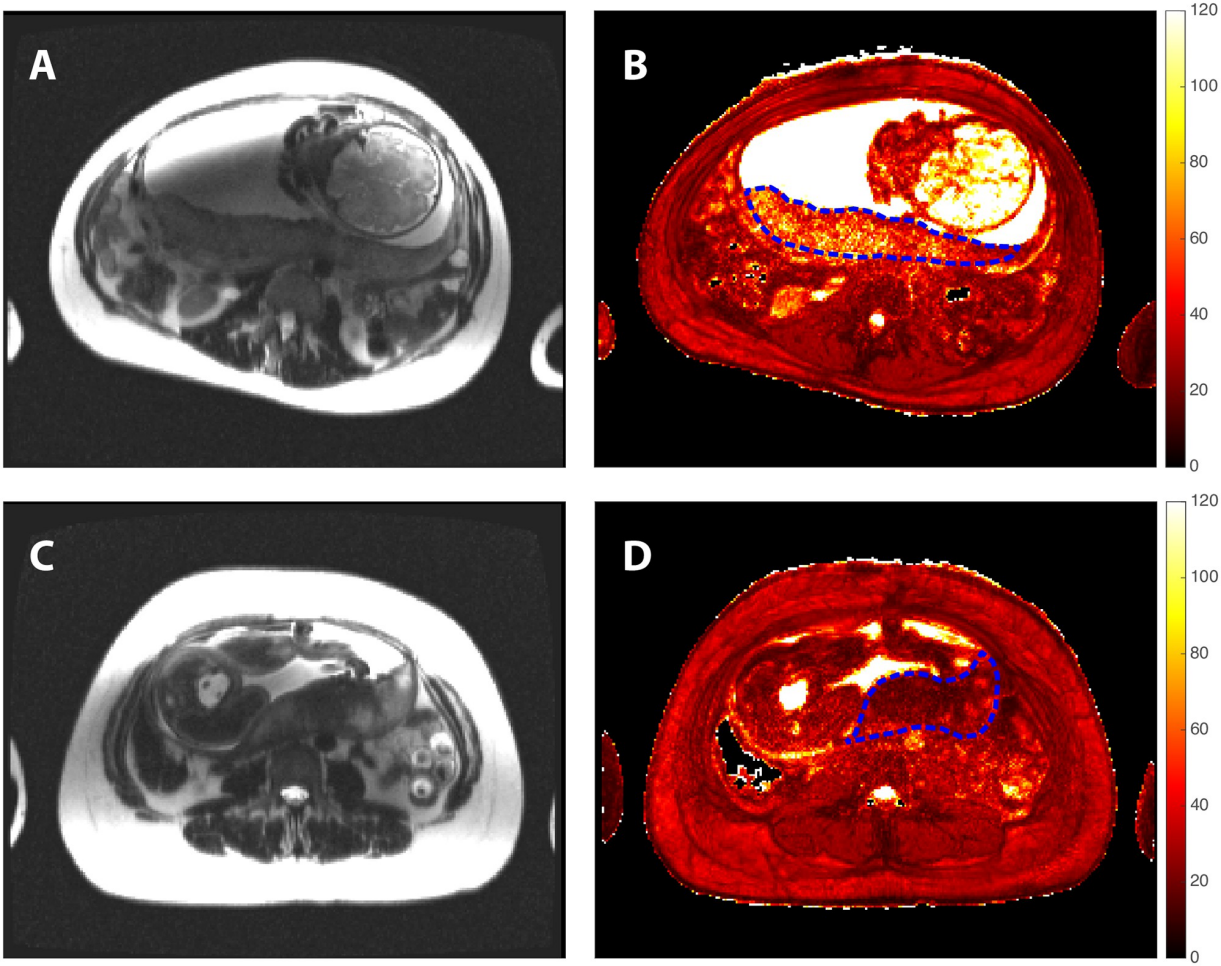
Comparison of anatomic magnetic resonance imaging and placental T2* mapping in uncomplicated normal and primary adverse pregnancies. T2-weighted HASTE MRI (left column) and placental T2* maps (right column) are shown for an uncomplicated normal pregnancy at 232 days gestation (top row, panels A & B) and for a primary adverse pregnancy at 235 days gestation presenting with severe preeclampsia (bottom row, panels C & D). The placenta is indicated by the dashed blue outlines overlaid on the T2* maps.

Fig 3 shows representative anatomic T2-weighted HASTE (left column) and quantitative T2* maps (right column) acquired in two study participants, matched for gestational age at time of scan. Placental ROIs are superimposed on the T2* maps (blue dashed lines). The upper row in the figure shows a UN pregnancy at 232 days of gestation with median placental T2* (= 51 ms) close to the population median (50th percentile), while the bottom row shows corresponding images for a PA pregnancy at 235 days gestation with a median T2* (= 26 ms) in the 1st percentile. Depression of the placental T2* in the latter is clearly apparent in panel D.

### Z-scores and receiver operating characteristic (ROC) curves for T2*

Statistics for T2* z-scores, stratified by category (UN/PA/SA), site (OHSU/Utah), and gestational age window (10–20 weeks, 20–30 weeks, and 30+ weeks), are given in Table 5, where z-scores derived from nonlinear sigmoid regression to T2* measurements in UN pregnancies were used as the reference distribution. As expected, the distribution of z-scores for UN pregnancies is symmetrical and centered on zero (mean = 0.02, SD = 1.00). Z-scores for the PA pregnancies are relatively symmetrical but broader and with a significant left shift (mean = -0.92, SD = 1.49, p<0.001, d = 0.85), while the distribution for SA pregnancies is shifted leftward (mean = -0.43, SD = 1.34, p = 0.002, d = 0.41) and notably skewed, suggesting the possibility of two subpopulations within the latter data. Differences between UN and PA were significant for all three gestational age windows for the combined group, with large effect size at the two later time points and a medium effect size at the first time point. When only OHSU data were considered, differences were significant and effect size was large at all three time points, while the Utah data only showed a significant difference (with large effect size) at mid-gestation.

**Table 5:** Placental T2* z-scores, with p-values and effect sizes (Cohen’s d), for study subjects stratified by category, site, and gestational age (GA) range. Effect size is considered small (green cells) when p< = 0.05 and 0.2< = d<0.5, medium (orange cells) when p< = 0.05 and 0.5< = d<0.8, and large (red cells) when p< = 0.05 and d> = 0.8.

	median		p-value	Cohen d	median		p-value	Cohen d	median		p-value	Cohen d
	Both sites											
	UN vs. PA				UN vs. SA				PA vs. SA			
All GA	-0.02	-0.92	0.000	0.85	-0.02	-0.43	0.002	0.41	-0.92	-0.43	0.000	0.35
10–20 weeks GA	0.04	-0.78	0.000	0.66	0.04	0.02	0.167	0.01	-0.78	0.02	0.035	0.63
21–30 weeks GA	-0.02	-1.35	0.000	1.27	-0.02	-0.46	0.020	0.46	-1.35	-0.46	0.006	0.55
31+ weeks GA	-0.07	-1.03	0.000	1.02	-0.07	-0.15	0.080	0.09	-1.03	-0.15	0.071	0.65
	OHSU only											
	UN vs. PA				UN vs. SA				PA vs. SA			
All GA	0.20	-1.46	0.000	1.47	0.20	0.02	0.027	0.20	-1.46	0.02	0.000	0.99
10–20 weeks GA	0.36	-1.08	0.000	1.29	0.36	0.13	0.568	0.20	-1.08	0.13	0.003	0.87
21–30 weeks GA	0.24	-1.84	0.000	1.77	0.24	-0.34	0.074	0.62	-1.84	-0.34	0.003	0.76
31+ weeks GA	-0.05	-1.51	0.000	1.44	-0.05	-0.43	0.129	0.47	-1.51	-0.43	0.025	0.84
	Utah only											
	UN vs. PA				UN vs. SA				PA vs. SA			
All GA	-0.17	-0.63	0.035	0.51	-0.17	-0.77	0.011	0.63	-0.63	-0.77	0.858	0.10
10–20 weeks GA	-0.24	-0.73	0.226	0.43	-0.24	-1.12	0.101	0.75	-0.73	-1.12	0.235	0.30
21–30 weeks GA	-0.24	-1.11	0.007	0.92	-0.24	-0.76	0.136	0.65	-1.11	-0.76	0.845	0.28
31+ weeks GA	-0.09	0.16	0.250	0.31	-0.09	0.31	0.348	0.45	0.16	0.31	0.909	0.15
	OHSU vs. Utah											
	UN				PA				SA			
All GA	0.20	-0.17	0.000	0.41	-1.46	-0.63	0.002	0.54	0.02	-0.77	0.032	0.59
10–20 weeks GA	0.36	-0.24	0.001	0.55	-1.08	-0.73	0.300	0.24	0.13	-1.12	0.022	0.74
21–30 weeks GA	0.24	-0.24	0.001	0.53	-1.84	-1.11	0.198	0.43	-0.34	-0.76	0.249	0.34
31+ weeks GA	-0.05	-0.09	0.340	0.05	-1.51	0.16	0.000	1.12	-0.43	0.31	0.394	0.63

**Fig 4:**
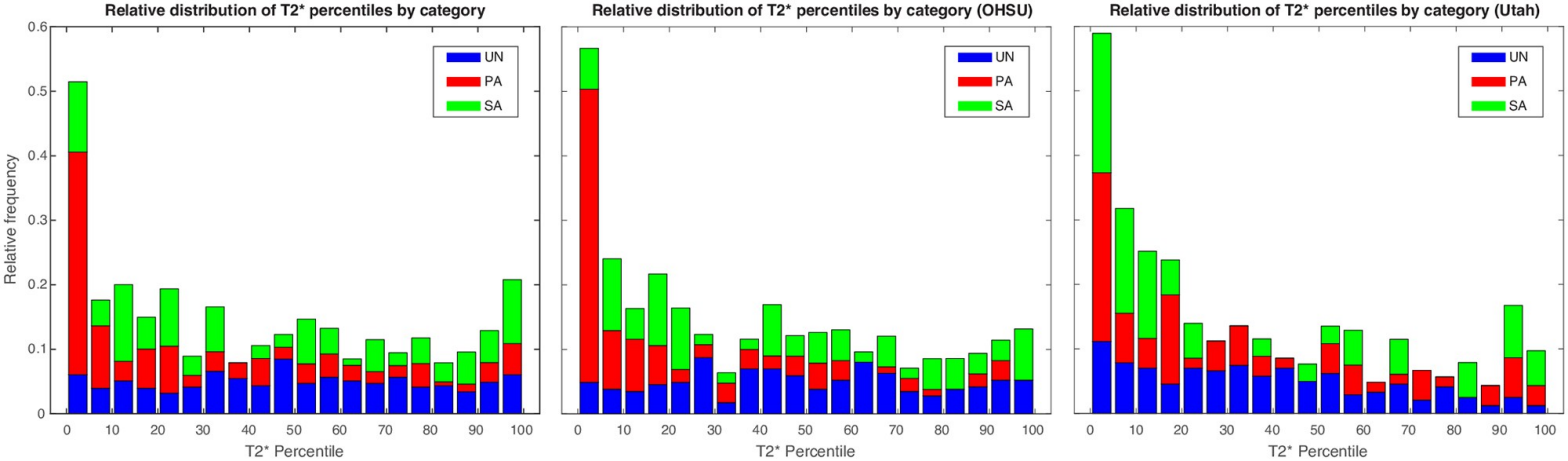
Histograms of T2* percentiles. Bar charts showing histograms of measured T2* percentiles for uncomplicated normal (UN, blue), primary adverse (PA, red), and secondary abnormal (SA, green) pregnancies for both sites (left) and for OHSU (middle) and Utah (right) separately.

The distributions of T2* percentiles derived from the z-score data are presented for both sites, and OHSU and Utah separately, in bar chart form in Fig 4, with twenty equally-spaced bins spanning from 0 to 100. The distribution of T2* percentiles in the UN population (blue) is, as expected, essentially uniform across the entire range, with roughly 5% of observations lying in each bin, while SA (green) pregnancies show modest enrichment at low values. In contrast, the PA pregnancies lie primarily in the lowest (0–5%) bin, with nearly 35% of the adverse studies lying in that range and 44% in the lowest 10% of T2* measurements. This effect is more pronounced in the OHSU data than in the Utah data; in the latter the low T2* values are more evenly apportioned between the PA and SA groups.

**Fig 5:**
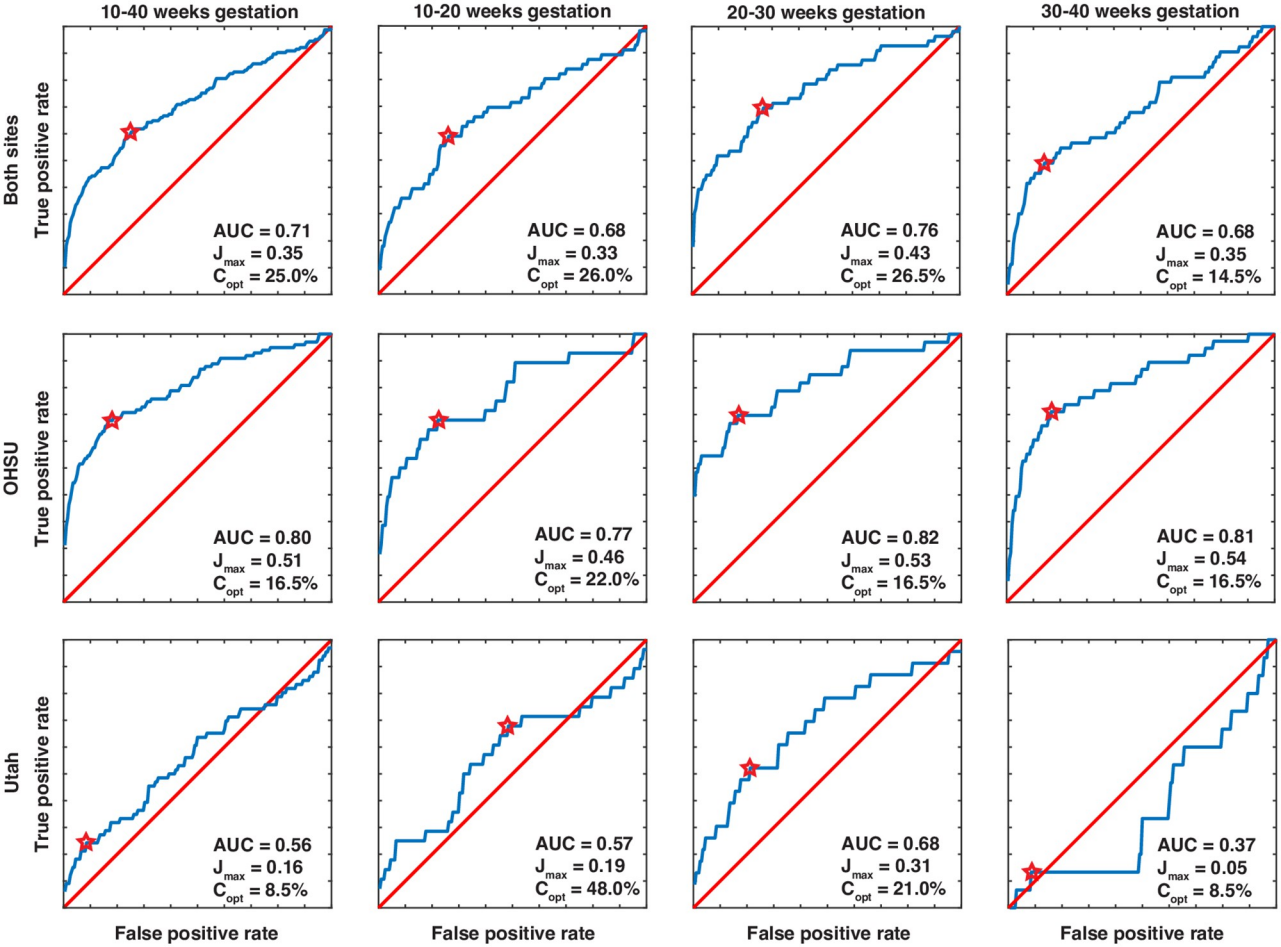
Receiver operator characteristic (ROC) curves for T2* prediction of PA pregnancies. The points where Youden’s J is maximized are indicated by the stars. Area under the curve (AUC), J_max_, and the corresponding optimal cutoff threshold in T2* percentile relative to UN (C_opt_) are given in the figure legend for each panel.

Fig 5 shows ROC curves for the entire population as well as stratified by gestational age window (10–20 weeks, 20–30 weeks, and 30+ weeks) and by site. For both sites across all gestational time points, the area under the curve (AUC) or C-statistic for placental T2* and PA pregnancy outcome is 0.71. The mid-gestation time period had the strongest predictive power (AUC of 0.76). C-statistics were consistently higher in the OHSU cohort than the Utah cohort, with the strongest C-statistic overall for OHSU studies at the mid-gestation time period (AUC = 0.82), and the weakest for Utah studies in late-gestation (AUC = 0.37).

### Placental T1

The longitudinal relaxation time of water, T1, is a central parameter in MRI. In order to investigate the potential of using this quantity to assess placental function, quantitative T1 values were acquired (in OHSU participants only). These data for UN pregnancies showed linear decrease with gestation at an average rate of -26.9 ms/week from approximately 2200 ms at 12 weeks gestation to roughly 1600 ms at 35 weeks (Table 5 and S3 Fig). Neither SA nor PA pregnancies showed any statistically significant differences in the evolution of T1 during pregnancy relative to UN, suggesting that placental T1 is not a useful metric for characterization of placental dysfunction.

### Placental volume

Placental volume was computed for all studies by computing the product of integrated number of voxels within the placental ROI with voxel volume, and was found to vary linearly with gestational age (Table 5 and S4a and S4b Fig), consistent with previous observations [50, 51]. No significant differences were observed between sites, but the placental volume for the PA group was significantly smaller than the UN group from 21 weeks onward.

### Maternal hemoglobin and oxygen saturation

Maternal hemoglobin level decreased linearly throughout gestation in UN pregnancies at an average rate of -0.046 mmol/week and was significantly higher in the Utah cohort than the OHSU cohort (mean difference 1.04±0.90 mmol, p<0.001, d = 0.90). Maternal hemoglobin was significantly higher in the PA pregnancies compared to UN (mean difference 0.36±1.04 mmol, p = 0.005, d = 0.34). There was no difference in hemoglobin between UN and SA pregnancies (Table 5 and S5a and S5b Fig).

Maternal SpO_2_ in UN pregnancies was found to be essentially constant throughout gestation (mean 97.0%) but was significantly lower in the Utah cohort than the OHSU cohort (mean difference -2.0±3.0%, p<0.001, d = 1.36). Neither SA nor PA pregnancies were associated with statistically different maternal SpO_2_ values or trends relative to UN (Table 5 and S6a and S6b Fig).

### Regression modeling results

Model definitions, best fit parameter values, fit parameter uncertainties, and root-mean-square (RMS) residual errors for regressions to all data and subsets discussed above are given in Table 4.

## Discussion

In this study, we characterized and modeled placental T2* across gestation in a large cohort of uncomplicated pregnancies. After establishing the normal evolution of T2* in UN pregnancies, we then demonstrated that median placental T2* is markedly lower in our adverse pregnancy outcome group. Pregnancies complicated by PA had lower T2* across gestation and had a larger rate of decline in early and mid-gestation when compared to UN pregnancies. This difference is persistent across gestation and occurs prior to clinical diagnosis of adverse outcome. The placenta is a dynamic organ which evolves over the entire course of gestation and adaptively develops in concert with the growing fetus. As a result, it is not a *fait accompli* that poor placental function early in gestation persists throughout pregnancy. However, our results suggest that even data from the early gestational window (10–20 weeks) demonstrate potential for identification of at-risk pregnancies.

Overall, we observed similar findings across our two independent sites, demonstrating that this method is robust and has the potential to be transferable across different institutions. Nevertheless, some relevant site-specific differences were observed that merit further clarification. We measured higher maternal hemoglobin level in Utah participants when compared to OHSU participants. Concordantly, participants had lower maternal oxygen saturation in Utah compared to OHSU. The lower maternal oxygen saturation is expected given the increased altitude in Salt Lake City, Utah when compared to Portland, Oregon, and the elevated maternal hemoglobin is compensatory to increase oxygen carrying capacity in a lower PO_2_ environment Although understanding of alterations in pregnancy physiology at high altitude is incomplete, there is evidence that multi-generational exposure leads to fetal and placental adaptation, including overall smaller fetal and neonatal size even in non-pathologic pregnancies [52–54]. There is evidence of placental adaptation with altered villous morphology and density at altitude, thought to be an attempt to make the most of what PO_2_ is available in the intervillous space [55]. One possible outcome of this is the lower median birthweight percentile in UN pregnancies at Utah (42.0), whereas at OHSU it was 50.5, suggesting a population shift to the left. It may be that altitude specific norms are necessary for optimal predictive power of T2* given these shifts in placental morphology and oxygenation, despite incorporation of maternal Hgb and SO_2_ into the modeling of T2*.

The ability of T2* measurements to discriminate between UN pregnancies and PA outcome pregnancies was much higher in the OHSU cohort than for Utah (AUC 0.80 vs 0.56). We suspect that this is due to site-specific differences in the prevalence of SGA and preeclampsia with severe features in this cohort, both of which are relatively under-represented in the Utah group. As noted above, in the Utah cohort, birthweights of neonates in the PA group were not statistically different than in UN pregnancies. Given that SGA and hypertensive diseases of pregnancy have multiple pathophysiologies with varying degrees of placental dysfunction, it is reasonable to propose that T2* quantification primarily identifies pathways linked to abnormalities attributable to perturbations of maternal placental blood flow and/or fetal oxygen uptake. It is possible that there is a secondary contribution due to the somewhat higher measurement error in the Utah data set as compared to OHSU, although the absolute measurement uncertainties are small for both study sites. Unfortunately, the modest number of PA pregnancies in our data set limits statistical power and precludes separation of the PA group into sub-categories. Moreover, it is also possible that our MRI pipeline is generalizable to multiple sites but requires altitude specific percentile norms to have optimal predictive strength. This is worth further investigation.

The imaging methodology in this study is highly amenable to clinical translation. Placental MRI was performed using imaging protocols and pulse sequences that are available on virtually all modern MRI scanners, and analysis of these data requires only minimal post-processing to convert signal measurements to T2* values. This protocol also avoids the need for gadolinium-based contrast agents which are otherwise the gold-standard for MRI assessment of tissue perfusion in non-pregnant diagnostic imaging. With the abundance of caution required for pregnant women, a methodology that avoids use of any exogenous contrast agent is highly favorable. In addition, because placental T2* is sensitive to the balance between oxygenated maternal blood delivery and fetal oxygen demand, it is particularly well-suited to identify problems stemming from inadequate placental oxygenation.

We acknowledge that both the PA clinical outcome group and the SA clinical outcome group definitions are imperfect, as is so often true in clinical obstetrics where we are dealing with syndromes rather than diseases. The PA clinical composite outcome was developed pragmatically. Gestational hypertensive disease, preeclampsia, low birth weight, and fetal death/stillbirth are all linked to placental dysfunction [3, 56, 57]. Although there are multiple pathways to each of these outcomes, some of which are not secondary to placental insufficiency, this composite was chosen because it is clinically meaningful when attempting to capture major morbidity and mortality due to placental insufficiency. Moreover, there is evidence of placental development abnormalities in the setting of some cases of spontaneous preterm birth. However, the pathophysiology of spontaneous preterm birth is thought to at least in part, be different than the pathophysiology of placental insufficiency [58]. Thus, we did not include spontaneous preterm birth in the PA group but rather in the SA group.

### Study strengths and limitations

Our study has a number of strengths. It is the largest prospective study of MRI assessment of placental function and the most extensive study of T2*. In addition, the longitudinal design enabled us to characterize the nonlinear evolution of T2* across pregnancy and provide reference values for both T2* itself, and rate of change in T2* within individual pregnancies as gestation progresses. While previously reported studies of changes in T2*-weighted BOLD-EPI measurements in response to hyperoxygenation demonstrate data acquisition efficiency, they are generally semi-quantitative, introduce methodological complexity, and alter the physiologic mechanisms that determine normal oxygen transport across a gradient [59–61]. In contrast, quantitative measurements of T2* are reflective of the balance between maternal delivery of oxygen and fetal demand and do not need ancillary experimental perturbations. Both the PA and SA composite outcomes we developed were defined prior to, and independent of, MRI data analysis. Both designation of pregnancy outcome and MRI data processing were blinded to each other and conducted independently prior to statistical analysis. By utilizing common, commercially available MRI acquisition protocols, the work described here is reproducible at other institutions, facilitating its potential use both in future clinical studies and in clinical practice.

There are also a number of limitations to this study. Although it is the largest longitudinal MRI study in pregnancy performed to date, the number of adverse outcomes was small (n = 70) and thus we were unable to stratify our composite outcome by individual diagnosis. Our study population is relatively ethnically and racially homogeneous, so the conclusions drawn may not be applicable to other populations. MRI was performed using 3 Tesla scanning hardware to increase sensitivity to changes in T2*, but these systems are not currently the standard in obstetric imaging and are not as widely available as 1.5 Tesla systems. While we used consistent criteria encompassing many common prenatal complications, there is no universally accepted definition of placental dysfunction or insufficiency. Moreover, the pathophysiology leading to clinical placental dysfunction is heterogenous by nature. In particular, we have previously identified circumstances where pathology related to villous inflammation or malformation can cause elevated T2* [45] in the setting of adequate supply of maternal arterial blood to the placenta in conjunction with impaired trans-villous oxygen permeability, which could constitute a confounding factor in some pregnancies. As a result, further refinement may be required to detect abnormally high, as well as abnormally low T2*, to accurately capture different types of abnormal placental development and function.

## Conclusion

This large, prospective longitudinal human study demonstrates the potential of quantitative T2* mapping during pregnancy to identify increased risk for adverse obstetric outcome due to placental dysfunction. We describe a data acquisition and processing pipeline that is reproducible and generalizable. Improved non-invasive diagnostics to identify pregnancies at risk of adverse outcomes due to placental dysfunction may facilitate discovery of novel biomarkers, improved stratification of patients in clinical studies, and allow for modification of clinical management plans.

## Supporting information

S1 Fig Histograms of number of MRI studies by gestational age. Stratified by category (S1a Fig) and site (S1b Fig).
(PDF)

S2 Fig Median T2*. Stratified by fetal sex for UN pregnancies (S2a Fig), maternal age for UN pregnancies (S2b Fig), maternal pre-pregnancy BMI for UN pregnancies (S2c Fig), site (OHSU vs. Utah) for PA pregnancies (S2d Fig), by mild vs. severe adversity for PA pregnancies (S2e Fig), and birthweight percentile for all pregnancies (S2f Fig). Sigmoid model regressions are plotted along with 95% CI and PI.
(PDF)

S3 Fig Median T1 for OHSU studies. Stratified by category. Linear model regressions from Table 4 are plotted along with 95% CI and PI.
(PDF)

S4 Fig Placental volume. Stratified by category (S4a Fig) and site (S4b Fig). Linear model regressions from Table 4 are plotted along with 95% CI and PI.
(PDF)

S5 Fig Maternal hemoglobin level. Stratified by category (S5a Fig) and site (S5b Fig). Linear model regressions from Table 4 are plotted along with 95% CI and PI.
(PDF)

S6 Fig Maternal SpO2. Stratified by category (S6a Fig) and site (S6b Fig). Linear model regressions from Table 4 are plotted along with 95% CI and PI.
(PDF)
