# Supplementary figures and images for: Quantitative longitudinal T2* mapping for assessing placental function and association with adverse pregnancy outcomes across gestation
Source: PLoS One. 2022 Jul 19;17(7):e0270360. doi: 10.1371/journal.pone.0270360 (PMC9295947; doi:10.1371/journal.pone.0270360)

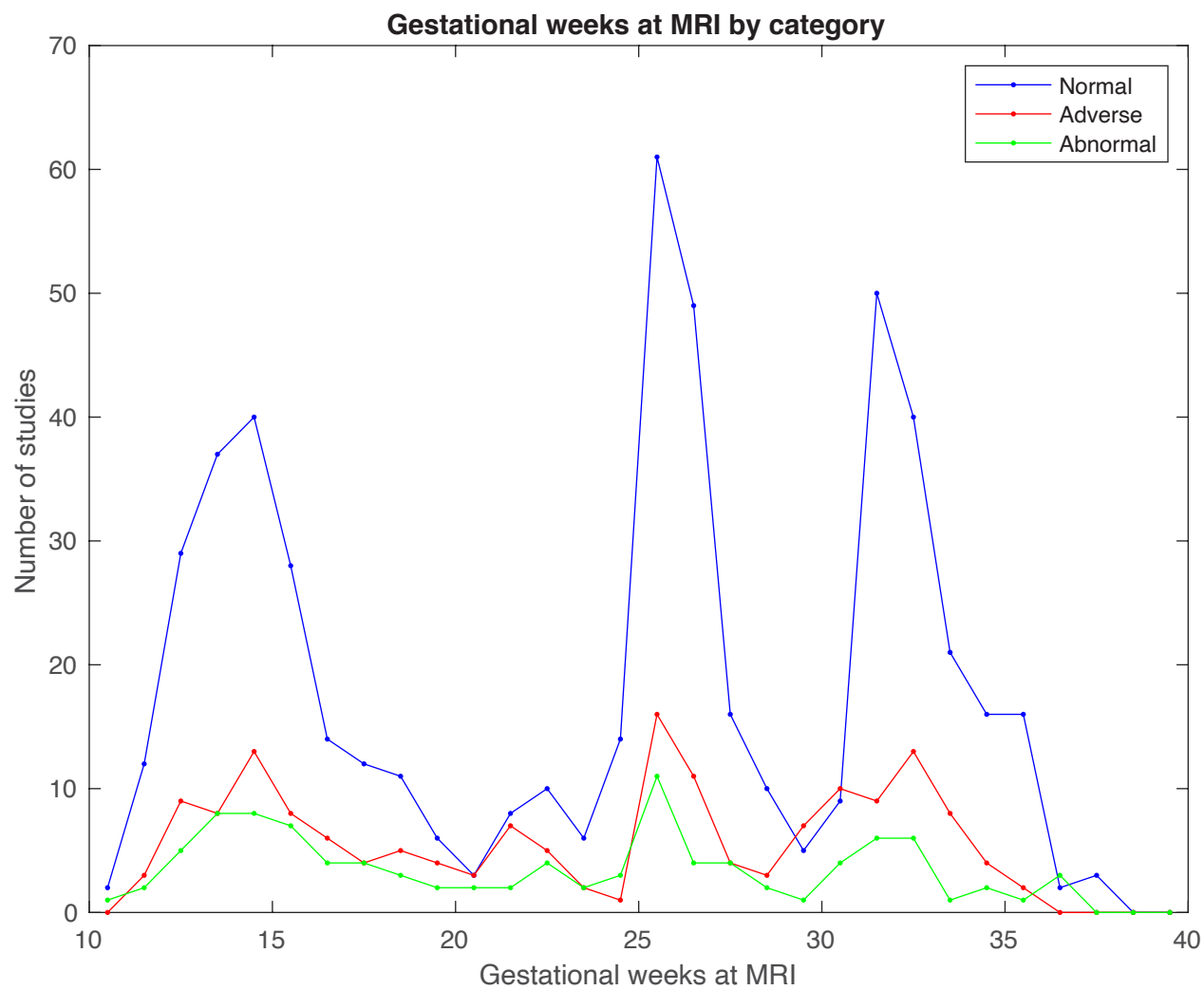

Figure S1a.

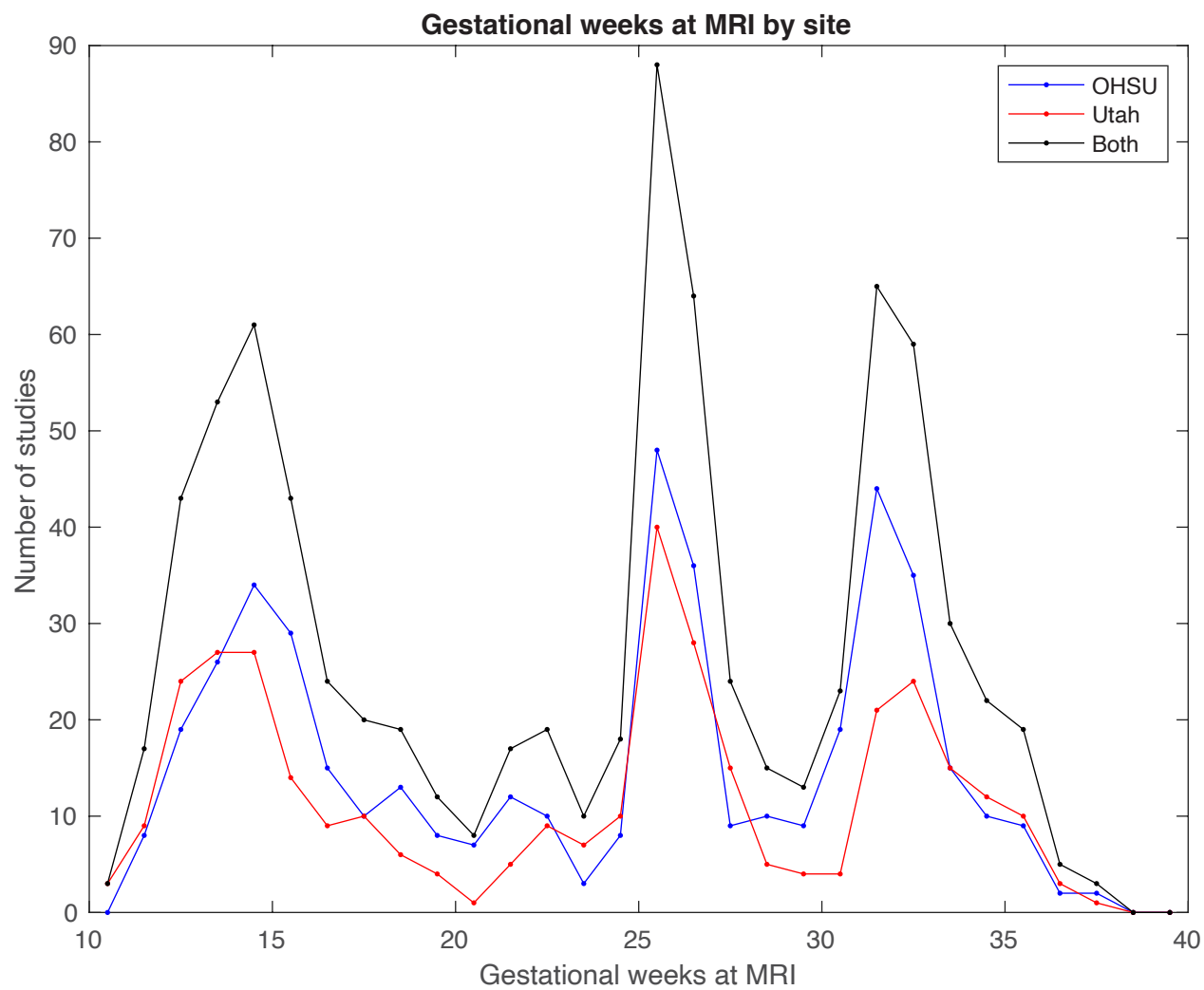

Figure S1b.

Supplement: S1 Fig — Stratified by category (S1a Fig) and site (S1b Fig). (PDF) [file pone.0270360.s001.pdf]

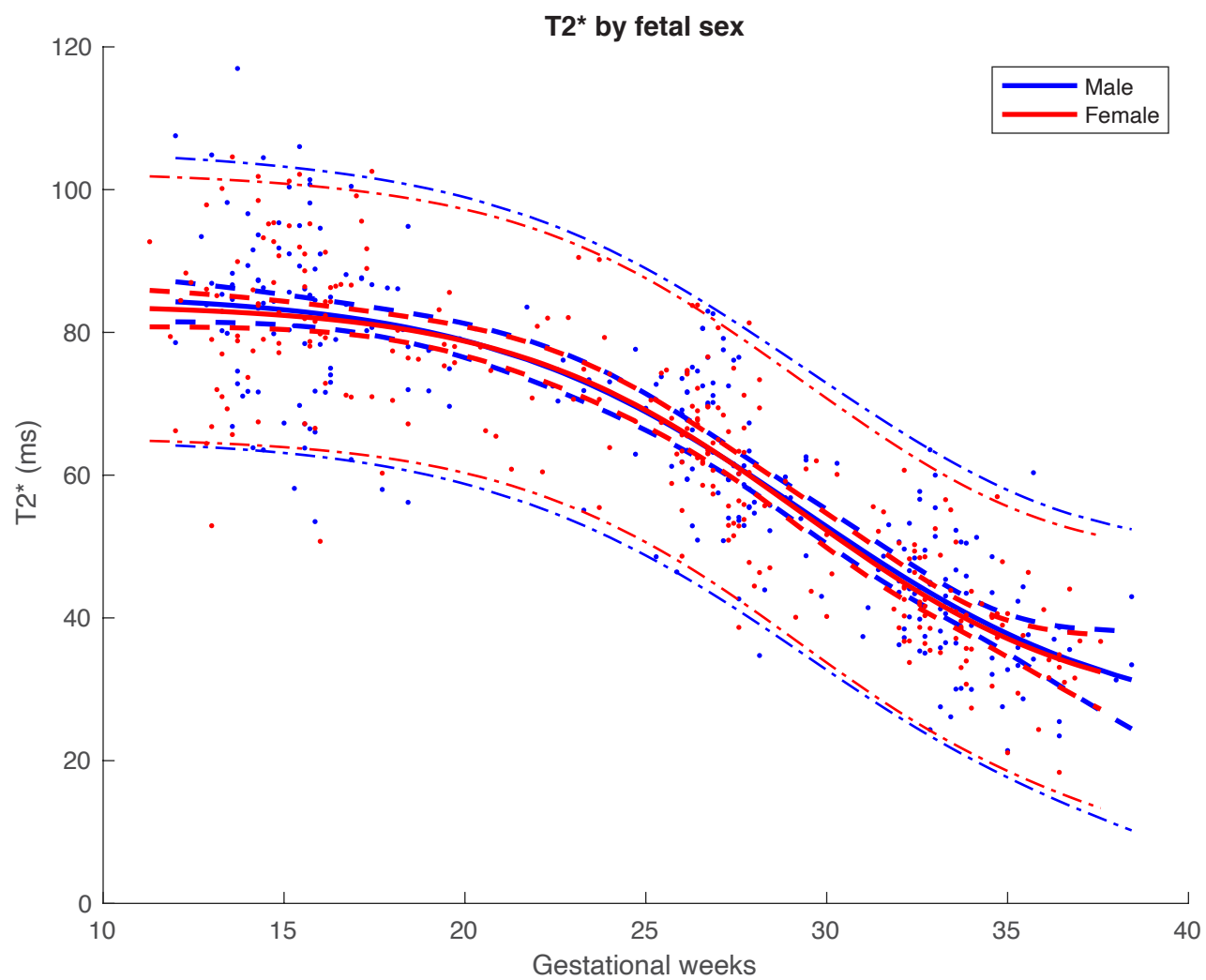

Figure S2a.

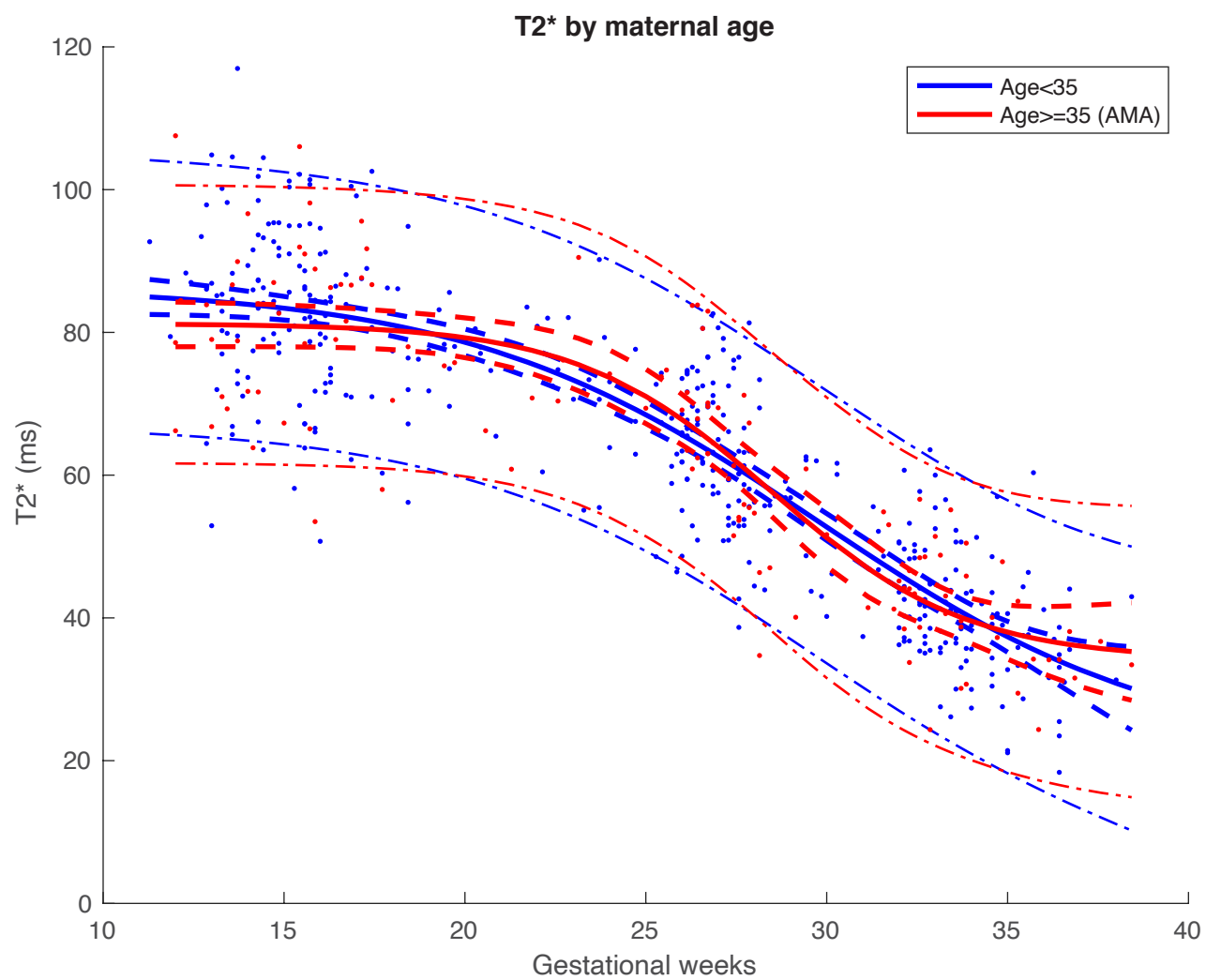

Figure S2b.

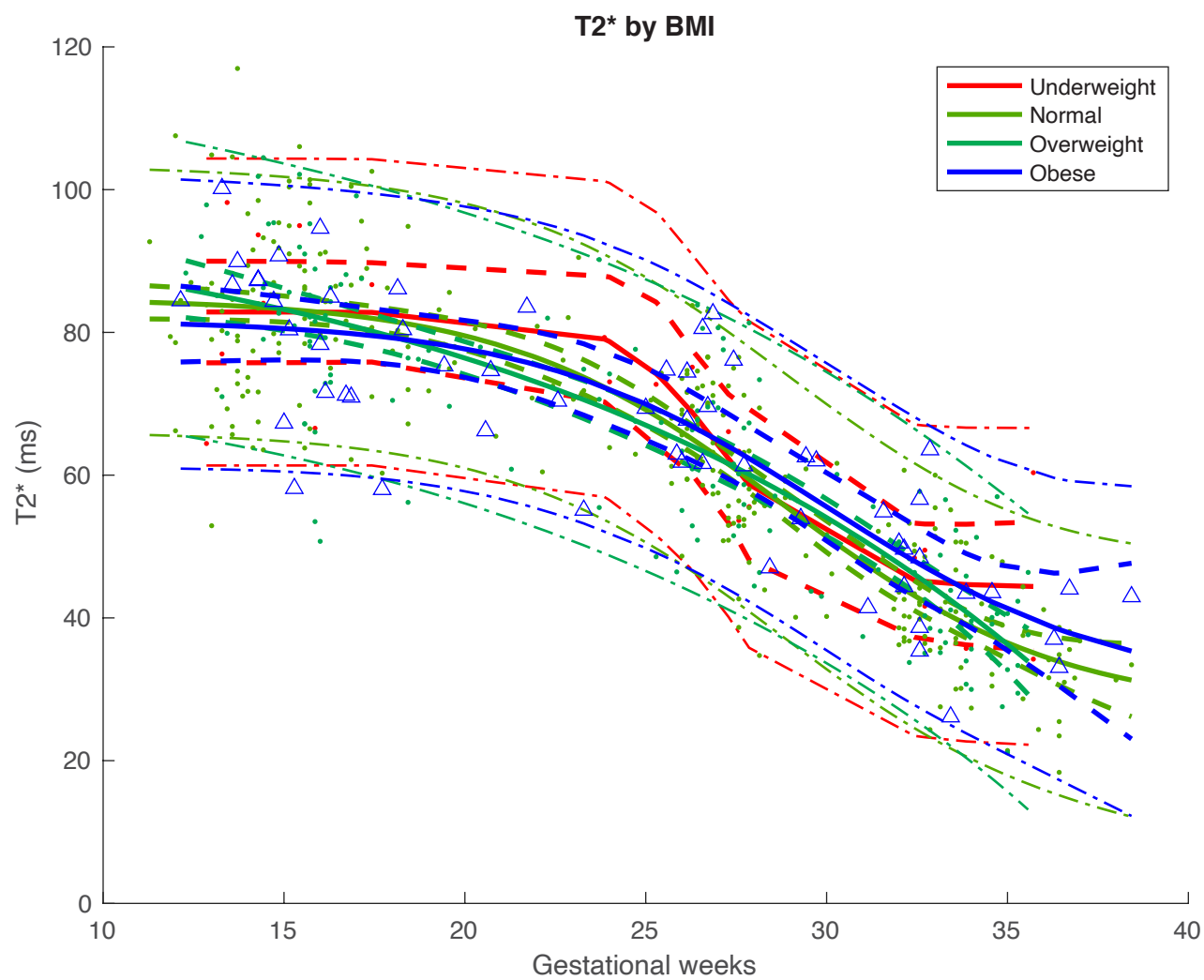

Figure S2c.

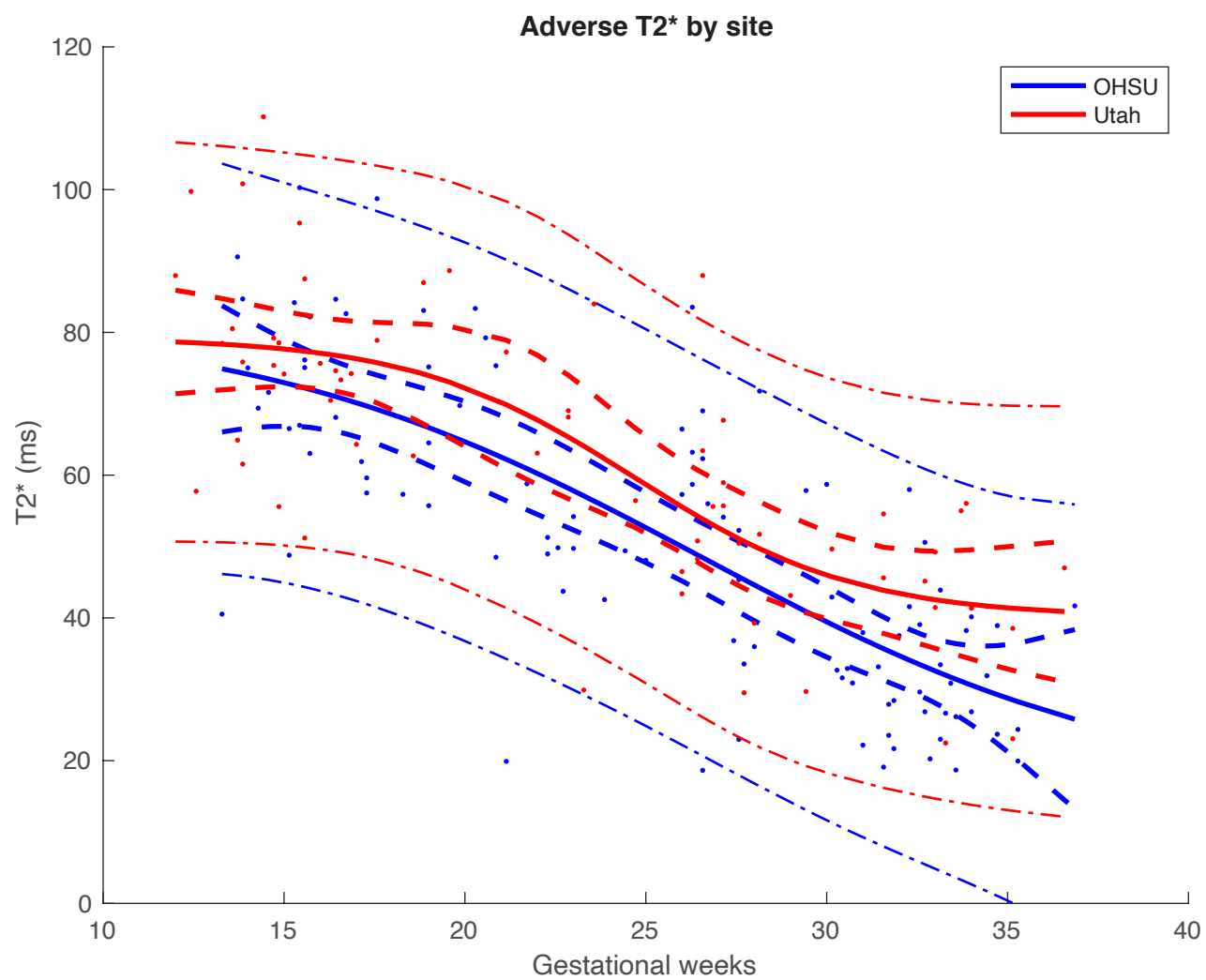

Figure S2d.

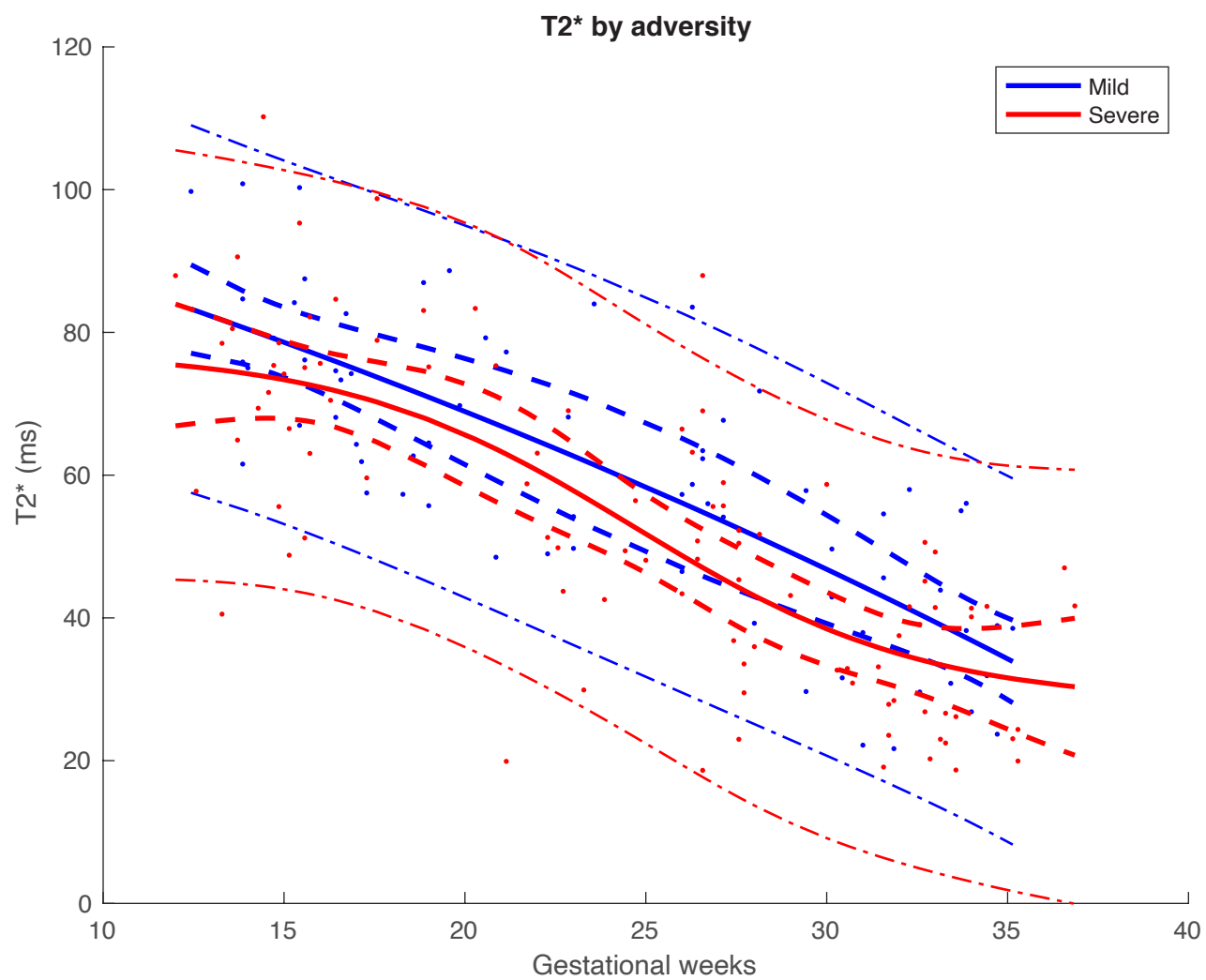

Figure S2e.

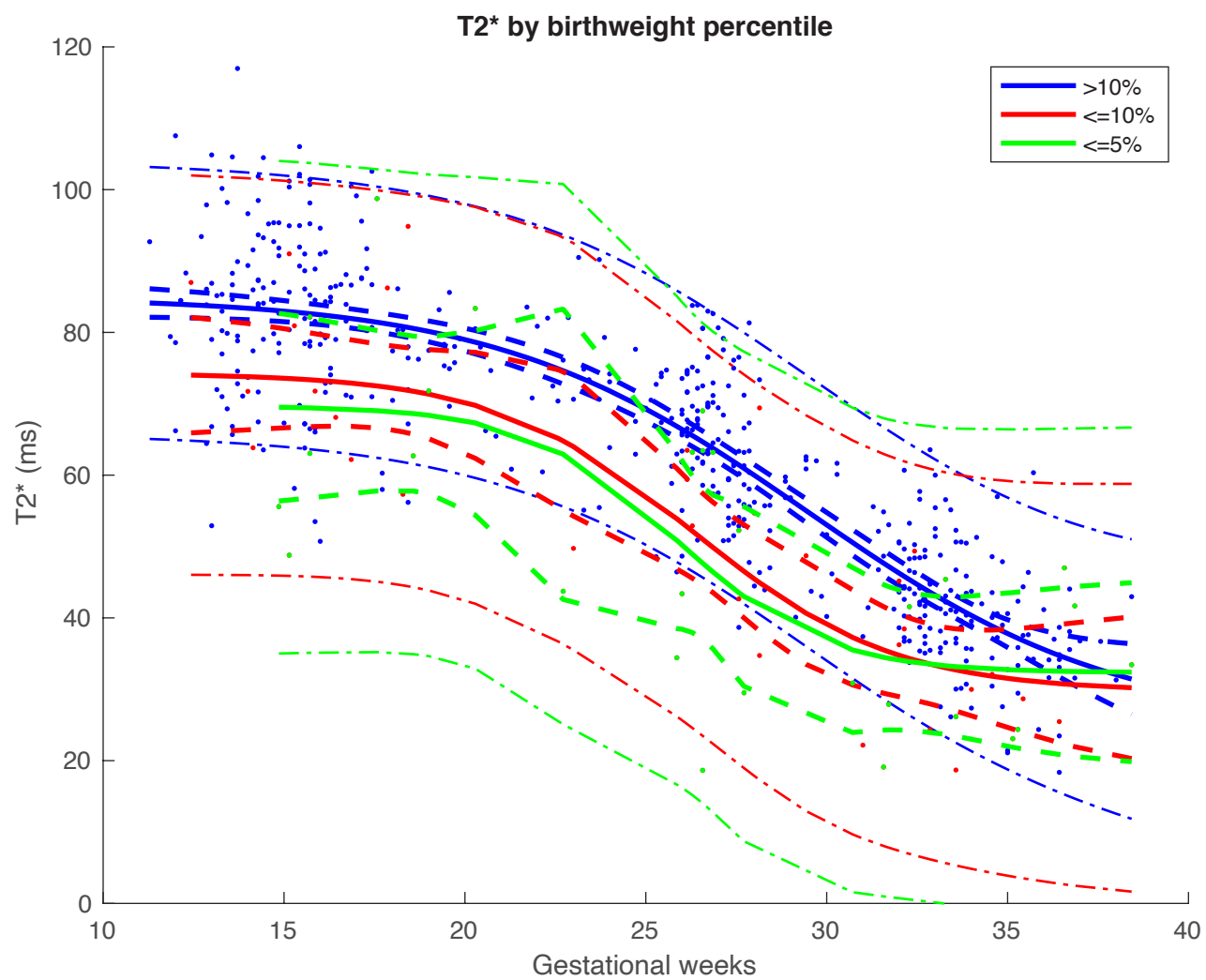

Supplement: S2 Fig — Stratified by fetal sex for UN pregnancies (S2a Fig), maternal age for UN pregnancies (S2b Fig), maternal pre-pregnancy BMI for UN pregnancies (S2c Fig), site (OHSU vs. Utah) for PA pregnancies (S2d Fig), by mild vs. severe adversity for PA pregnancies (S2e Fig), and birthweight percentile for all pregnancies (S2f Fig). Sigmoid model regressions are plotted along with 95% CI and PI. (PDF) [file pone.0270360.s002.pdf]

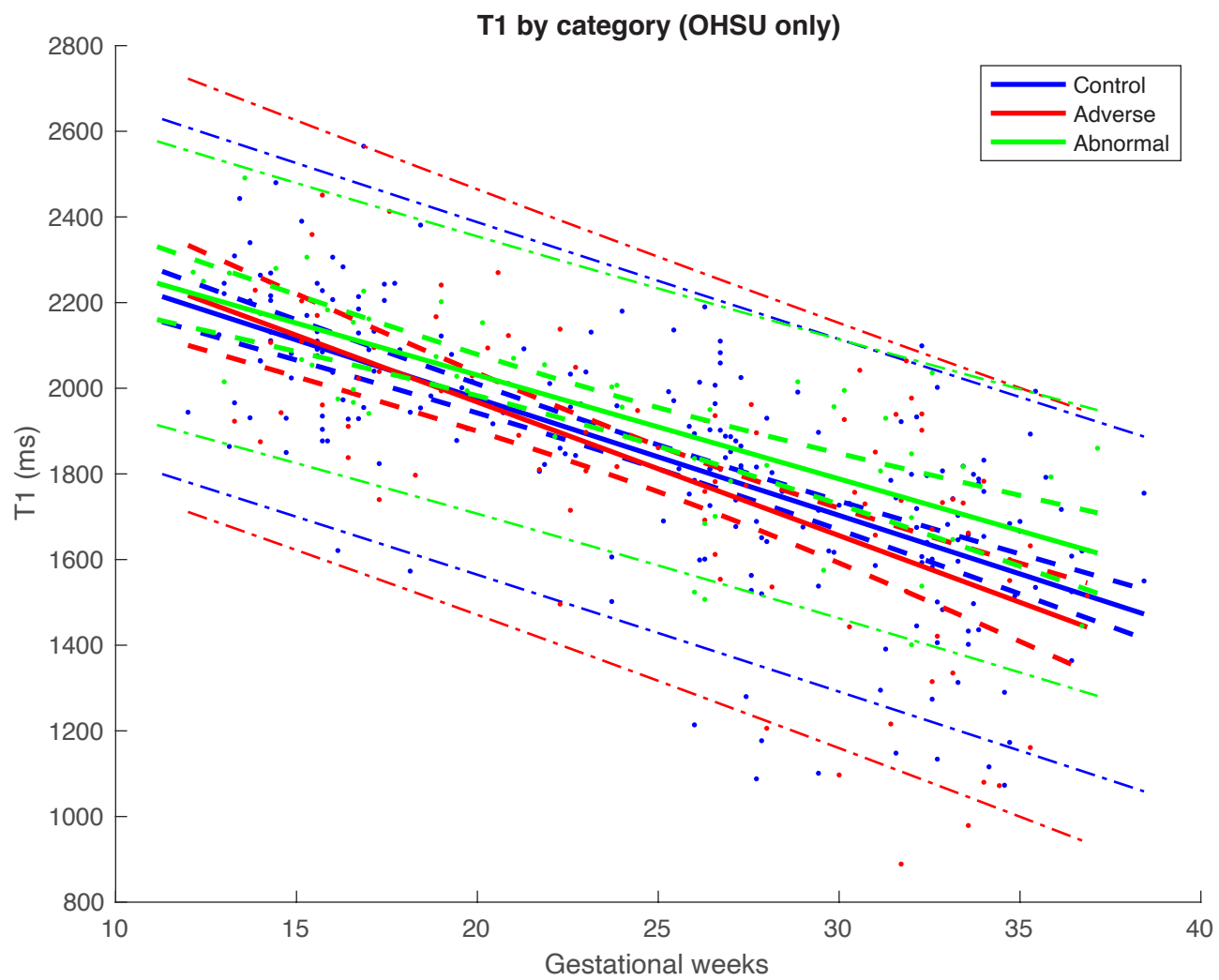

Figure S3.

Supplement: S3 Fig — Stratified by category. Linear model regressions from Table 4 are plotted along with 95% CI and PI. (PDF) [file pone.0270360.s003.pdf]

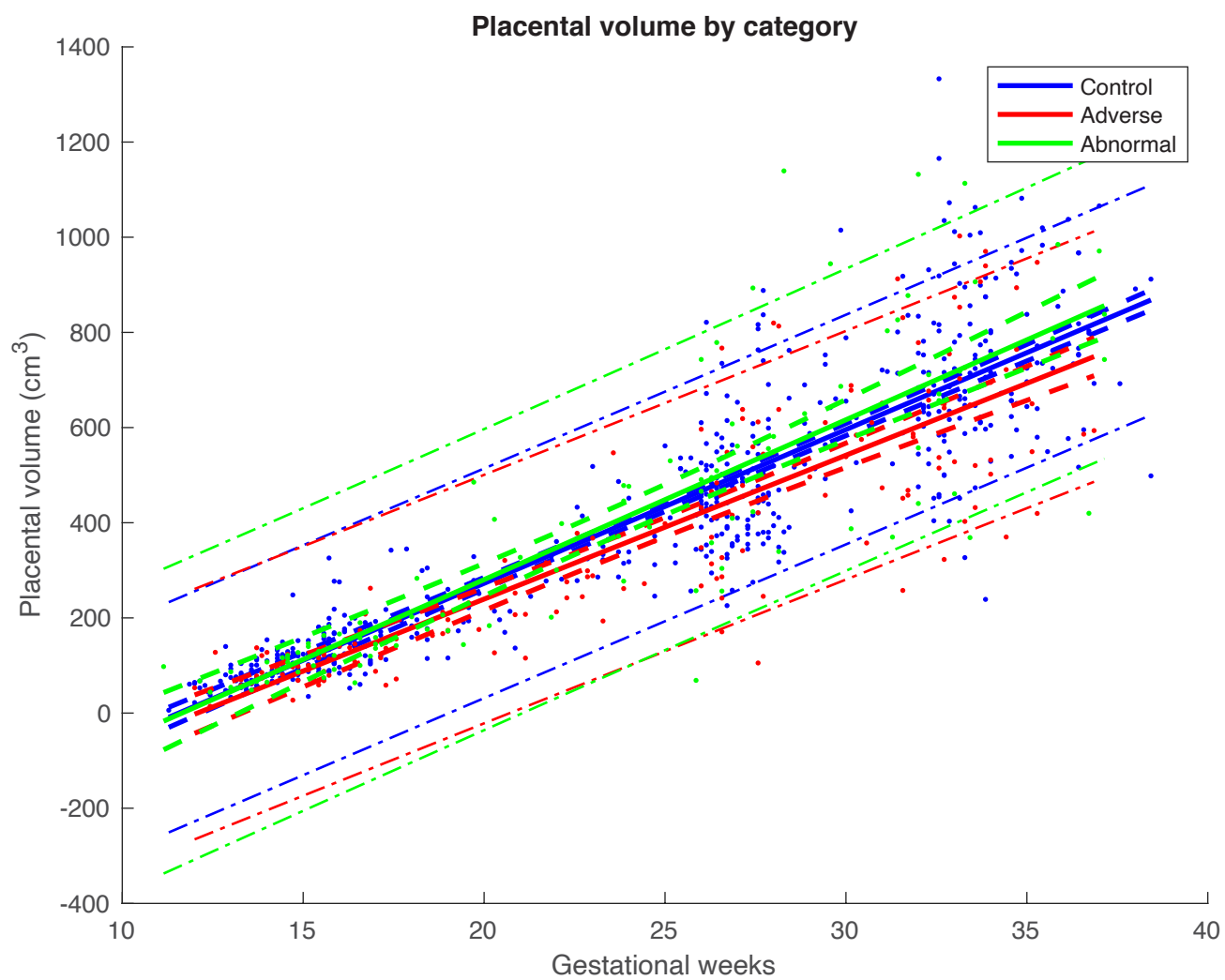

Figure S4a.

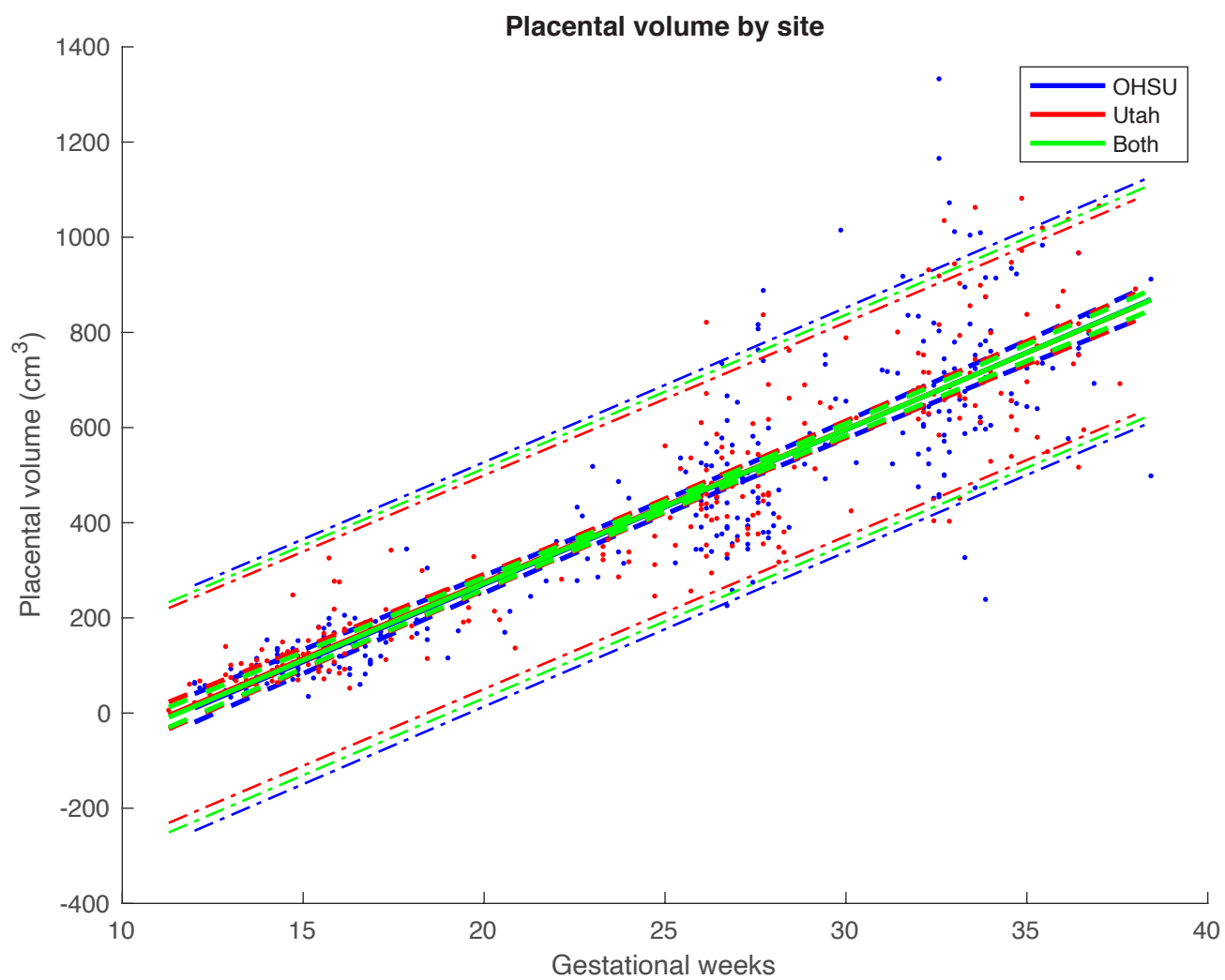

Figure S4b.

Supplement: S4 Fig — Stratified by category (S4a Fig) and site (S4b Fig). Linear model regressions from Table 4 are plotted along with 95% CI and PI. (PDF) [file pone.0270360.s004.pdf]

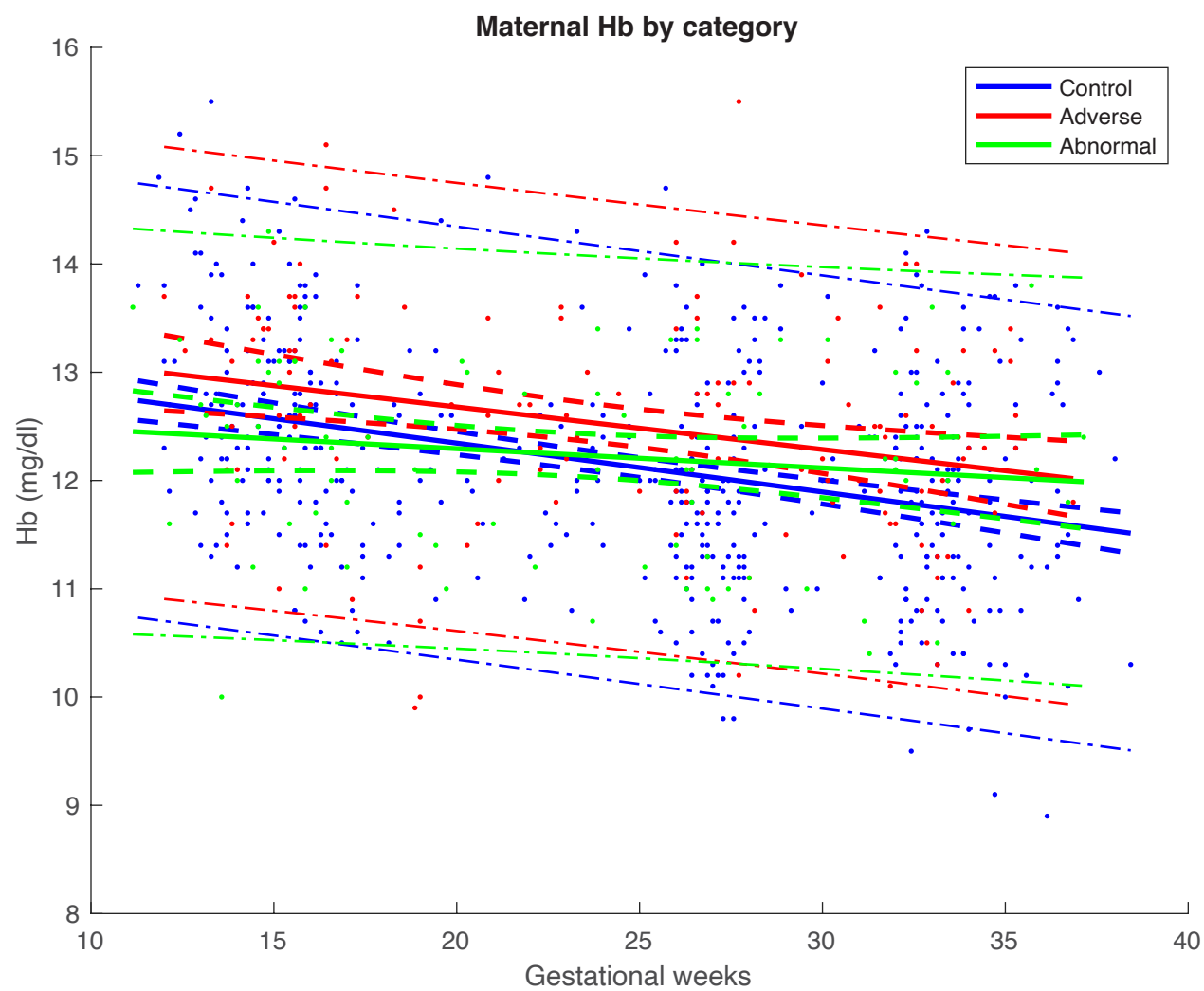

Figure S5a.

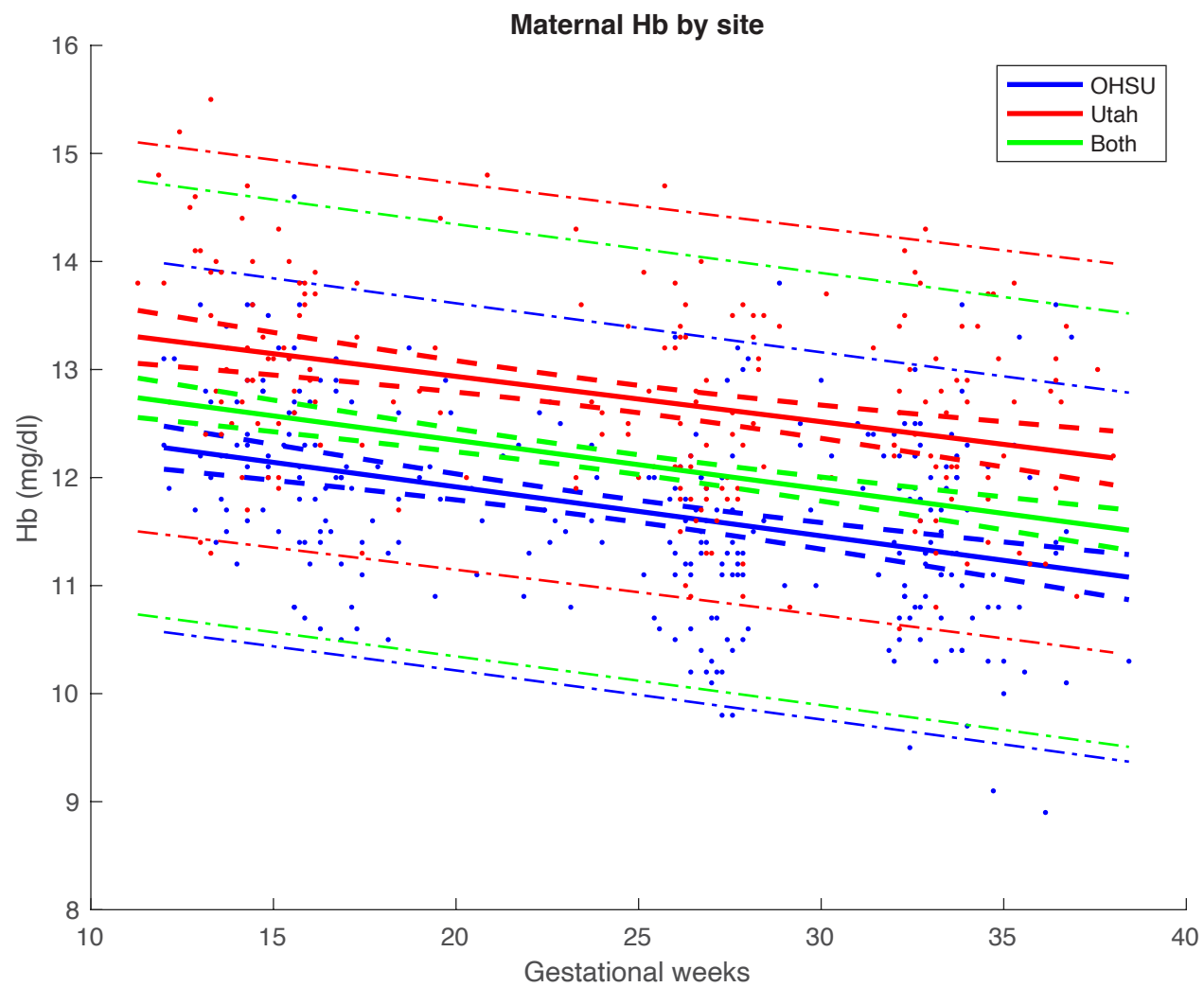

Figure S5b.

Supplement: S5 Fig — Stratified by category (S5a Fig) and site (S5b Fig). Linear model regressions from Table 4 are plotted along with 95% CI and PI. (PDF) [file pone.0270360.s005.pdf]

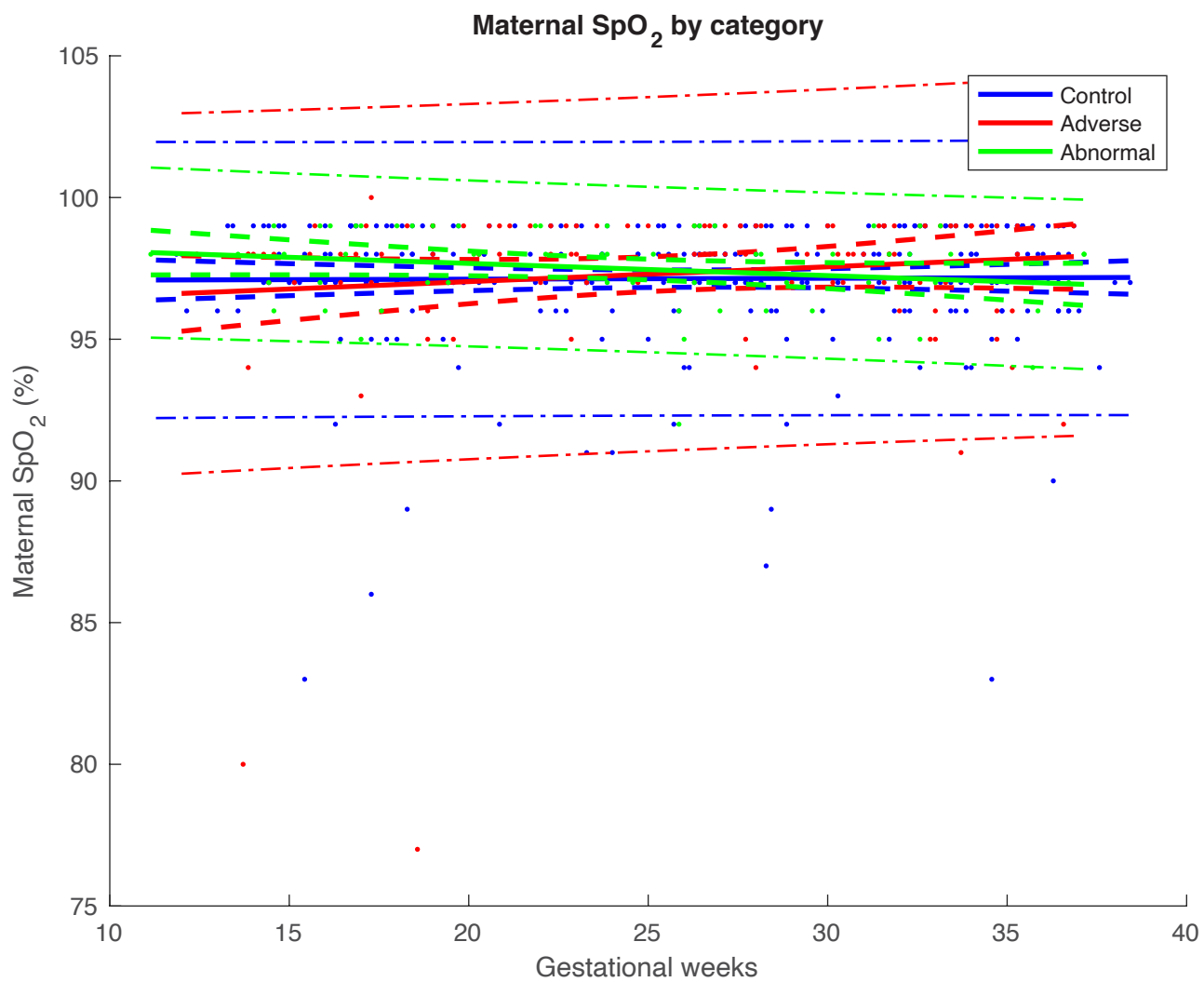

Figure S6a.

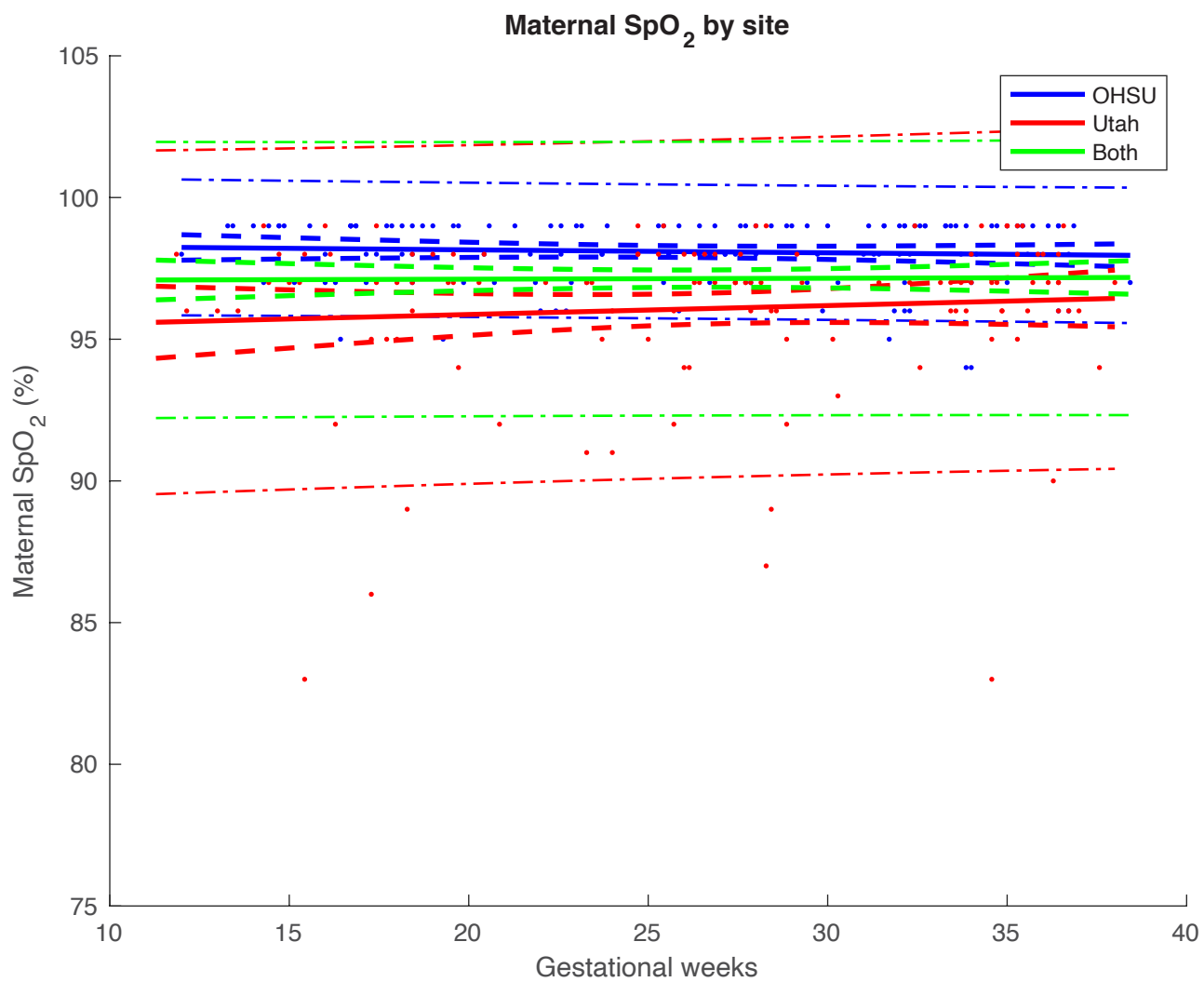

Figure S6b.

Supplement: S6 Fig — Stratified by category (S6a Fig) and site (S6b Fig). Linear model regressions from Table 4 are plotted along with 95% CI and PI. (PDF) [file pone.0270360.s006.pdf]
